# Natural selection in a population of *Drosophila melanogaster* explained by changes in gene expression caused by sequence variation in core promoter regions

**DOI:** 10.1186/s12862-016-0606-3

**Published:** 2016-02-09

**Authors:** Mitsuhiko P. Sato, Takashi Makino, Masakado Kawata

**Affiliations:** Department of Ecology and Evolutionary Biology, Graduate School of Life Sciences, Tohoku University, 6-3, Aramaki Aza Aoba, Aoba-ku, Sendai, 980-8578 Japan

**Keywords:** Core promoter region, Natural selection, Population genetics, Transcriptomics

## Abstract

**Background:**

Understanding the evolutionary forces that influence variation in gene regulatory regions in natural populations is an important challenge for evolutionary biology because natural selection for such variations could promote adaptive phenotypic evolution. Recently, whole-genome sequence analyses have identified regulatory regions subject to natural selection. However, these studies could not identify the relationship between sequence variation in the detected regions and change in gene expression levels. We analyzed sequence variations in core promoter regions, which are critical regions for gene regulation in higher eukaryotes, in a natural population of *Drosophila melanogaster*, and identified core promoter sequence variations associated with differences in gene expression levels subjected to natural selection.

**Results:**

Among the core promoter regions whose sequence variation could change transcription factor binding sites and explain differences in expression levels, three core promoter regions were detected as candidates associated with purifying selection or selective sweep and seven as candidates associated with balancing selection, excluding the possibility of linkage between these regions and core promoter regions. *CHKov1*, which confers resistance to the sigma virus and related insecticides, was identified as core promoter regions that has been subject to selective sweep, although it could not be denied that selection for variation in core promoter regions was due to linked single nucleotide polymorphisms in the regulatory region outside core promoter regions. Nucleotide changes in core promoter regions of *CHKov1* caused the loss of two basal transcription factor binding sites and acquisition of one transcription factor binding site, resulting in decreased gene expression levels. Of nine core promoter regions regions associated with balancing selection, *brat*, and *CG9044* are associated with neuromuscular junction development, and *Nmda1* are associated with learning, behavioral plasticity, and memory. Diversity of neural and behavioral traits may have been maintained by balancing selection.

**Conclusions:**

Our results revealed the evolutionary process occurring by natural selection for differences in gene expression levels caused by sequence variation in core promoter regions in a natural population. The sequences of core promoter regions were diverse even within the population, possibly providing a source for natural selection.

**Electronic supplementary material:**

The online version of this article (doi:10.1186/s12862-016-0606-3) contains supplementary material, which is available to authorized users.

## Background

Understanding the evolutionary forces that influence genetic variation in natural populations is a fundamental issue in evolutionary biology. Recent studies have emphasized that the evolution of gene regulatory sequences is important for adaptive evolution [1] and thus, regulatory regions may play a major role in adaptation [2]. Several studies have shown that *cis*-regulatory mutations are involved in the evolution of phenotypic changes [2, 3]. Recent or current natural selection and adaptive evolution can be detected by various methods, such as nucleotide diversity (π) and Tajima’s *D* test for whole-genome DNA sequences and the McDonald–Kreitman test [4] for coding regions. Even for sequences of gene regulatory regions, several methods have been proposed and genome-wide analyses have also shown statistical evidence of natural selection in non-coding and *cis*-regulatory regions [5–7]. These studies focused on nucleotide substitutions in regulatory sequences or transcription factor binding sites (TFBSs) between species and did not evaluate recent or ongoing selection for standing genetic variation in natural populations. In addition, it is unknown how sequence differences affect the binding of transcription factors and regulate gene expression. Recently, analyses of human whole-genome variation data and genome-wide chromatin immunoprecipitation data have identified adaptive substitution and deleterious polymorphisms at TFBSs [8]. However, this study did not show how variations in regulatory sequences affected gene expression levels.

Variations in gene expression are considered to affect phenotypic consequences in morphology, physiology, behavior, and disease susceptibility [9, 10]. For this reason, among sequence variations in regulatory regions, those affecting gene expression are thought to be important for phenotypic variation. Transcriptomic technologies, such as microarray and high-throughput RNA sequencing (RNA-seq), make it possible to observe variation in gene expression in natural populations of species including humans [11–13], fish [14], mice [15], fruitfly [16–18], and yeast [19, 20]. These transcriptomic technologies provide evidence of adaptive differences among natural populations [17, 18, 21]. Thus, when data describing variation in whole-genome sequences and gene expression levels in natural populations are available, we can detect sequence variations in gene regulatory regions that cause gene expression variation that has been subject to natural selection.

For *Drosophila melanogaster*, genome and transcriptome data from inbred lines derived from a natural population in the state of North Carolina, USA, are stored in the *Drosophila* Genetic Reference Panel (DGRP), a community resource for analysis of population genomics [22]. The database contains whole-genome sequences of 168 individuals from a natural population. Gene expression data from transcriptome analysis of inbred lines from the same individuals in the population are also available [16]. In this study, we focused on core promoter regions (CPRs), which are critical regions for gene regulation in higher eukaryotes. CPRs are generally defined as DNA regions that direct the accurate initiation of transcription by RNA polymerase II and contain various sequence motifs (such as TATA box, BRE (TFIIB recognition element), Inr (Initiator), and DPE (Downstream promoter element)) that interact with basal transcription factors. The mechanism affecting expression levels is more clearly understood [23] for CPRs than for other complex regulatory regions. CPRs containing motifs on chromosomes in *D. melanogaster* [24] and sequence motifs that can contribute to activity by CPRs in eukaryotes [25] are available for analysis. Using these data, adaptive regulatory sequence variations that actually affect differences in gene expression levels can be detected.

In the present study, we examined sequence variation in CPRs in a natural population of *D. melanogaster*. Among variations associated with differences in gene expression levels, we identified those that have been subjected to natural selection. We also inferred differences in nucleotide sequences responsible for the gene expression differences.

## Results

### Detecting transcripts whose expression variation was explained by sequence variation

**Fig. 1:**
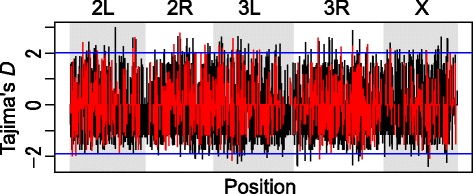
Distribution of Tajima’s *D* in core promoter regions along chromosome arms of *D. melanogaster*. Black vertical bars show Tajima’s *D* of core promoter regions (CPRs) for all of the transcripts used. Red vertical bars show Tajima’s *D* of transcripts in which expression variation was significantly explained by sequence variation in CPRs. Blue horizontal lines indicate critical values at which the Tajima’s *D* values of CPRs are significantly higher or lower than zero (*P* < 0.01), based on a null distribution generated by coalescent simulations

Genome and transcriptome data from a natural North American population of *D. melanogaster* was used to estimate the relative contribution of CPRs to changes in gene expression levels by sequence mutations in CPRs. Of the 11,454 known CPRs, 6799 were expressed with high broad-sense heritability and without minor alleles, and 6617 (97.32 %) did not contain undetermined nucleotides for 20 or more individual lines (see Methods). The average and median lengths of CPRs were 169.4 bp and 160 bp, and the average and median numbers of segregating sites were 3.26 bp and 2 bp. The average nucleotide diversity (π) and Watterson’s θ_w_ [26] were 0.00561 and 0.00608, respectively. For the population of 168 *D. melanogaster* individuals used in this study, π and Watterson’s θ_w_ over the entire genome were 0.0056 and 0.0067, respectively. The average π and θ_w_ values over whole coding sequences (CDS) for the entire genome were 0.0037 and 0.0040, respectively [22]. Sequence variation in CPRs was similar to that in the entire genome and higher than that in CDS in this natural population. Linear model analysis showed that among the 6617 expressed transcripts with high heritability and polymorphic sites and without undetermined nucleotides, 996 (14.65 %) expression variations were significantly associated with sequence differences in CPRs after Benjamini-Hochberg multiple-test correction (Fig. 1 and Additional file 1). Expression levels of 5429 transcripts (79.84 %) were significantly influenced by sex, and expression levels of 116 transcripts (1.71 %) were significantly influenced by sequence-by-sex interaction after multiple-test correction (Additional file 1). Sets of these genes whose expression levels were explained by sequence differences in CPR or sex were not enriched for any gene ontology (GO) functions.

### Natural selection on sequence differences in CPRs associated with variation in expression level

**Fig. 2:**
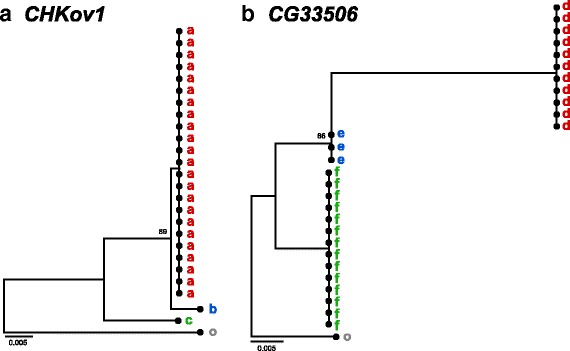
Phylogeny of different alleles of CPRs for *CHKov1* and *CG33506* found in the natural population. Neighbor-joining trees for different alleles (*a*-*c* and *d*-*f*) of CPR sequences are shown for *CHKov1* (**a**) and *CG33506* (**b**) found in a natural population of *D. melanogaster. D. simulans* was used as an outgroup (o). Bootstrap values are shown for nodes with > 60 % support

We identified CPRs associated with expression variation using a linear model and subjected them to natural selection by coalescent simulations. The coalescent simulations were conducting based on the demographic history of the North Carolina population [27] in which the genome and transcriptome data was obtained. CPRs for which Tajima’s *D* [28] value were significantly (*P* < 0.01) lower than zero based on the null distribution obtained by the coalescent simulations were considered as candidates under purifying selection or selective sweep, and those with significantly (*P* < 0.01) higher Tajima’s *D* value as candidates under balancing selection. The average Tajima’s *D* statistic of CPRs was −0.179. We detected eight CPRs associated with purifying selection or selective sweep and 23 CPRs associated with balancing selection. Two of eight candidates associated with purifying selection or selective sweep encoded the same transcript (*Sucb*) and two of 23 candidates associated with balancing selection encoded the same transcript (*CalpA*). They were identified by more than one probe in a microarray. In four of the candidates associated with purifying selection or selective sweep and nine candidates associated with balancing selection, sequence variation in CPRs could change TFBSs because position-specific scoring matrix (PSSM) scores (log odds scoring matrix, see Methods) differed across threshold values (Table 1 and Additional file 2). Among the sequence variation with different TFBSs, variation in CPRs that did not lead to differences in expression levels is excluded from Table 1. Phylogenetic trees of polymorphic alleles for CPRs with negative Tajima’s *D* values indicated that CPRs for *CHKov1* and *CG11590* had been subject to selective sweep, and *MBD*-*R2* and *CG17660* to purifying selection (Fig. 2a and Additional file 3).

**Table 1:** Names of genes for which sequence variations in CPRs were identified as outliers by Tajima’s *D* test and gene expression variation could be explained by sequence variation in CPRs

Gene name	FDR	π	Tajima’s *D*	# TFBSs	Linkage with neighbor
*CG33506*	1.84E-03	0.0262	2.776	3	N
*CG10463*	9.300E-08	0.0179	2.603	2	N
*CG6950*	8.286E-03	0.0123	2.578	7	N
*Nmda1*	2.969E-04	0.00649	2.335	1	Y
*CG14253*	2.602E-02	0.0124	2.326	1	N
*Cyp4d1*	1.240E-09	0.0151	2.175	1	Y
*brat*	1.541E-02	0.00583	2.171	1	N
*CG9044*	9.963E-03	0.0171	2.094	1	N
*CG15743*	4.214E-02	0.00607	2.074	2	N
*CG17660*	1.586E-04	0.00255	−1.958	4	N
*CG11590*	2.735E-02	0.00648	−2.081	1	N
*MBD*-*R2*	1.061E-02	0.00387	−2.098	2	N
*CHKov1*	2.437E-09	0.00249	−2.106	3	Y

Genes in which sequence variations in CPRs did not change TFBSs and different TFBSs did not affect differences in expression levels were excluded

*FDR* false discovery rates for the linear model used to detect the relationship between gene expression and sequence variations

# TFBSs = the number of TFBSs estimated by PSSM scores, which differed among the alleles of CPRs found in the population

Linkage with neighbor: linkage with one or more SNPs in noncoding regions flanking CPR could explain differences in expression level

Detected association between variation in CPRs and differences in expression levels could be due to linkage between CPR and single nucleotide polymorphisms (SNPs) in regulatory regions outside CPRs. In addition, natural selection for CPRs may be incorrectly inferred owing to linkage with a neighboring regulatory region that has been subject to selection. We accordingly investigated whether variation in CPRs detected as subject to selection was in linkage with SNPs associated with expression differences in non-coding regions flanking CPRs. For six genes (*CG15743*, *CG9044*, *brat*: brain tumor, *CG6950*, *CG10463*, and *CG33506*) detected as being under balancing selection, *CG11590* under positive selection, and *MBD*-*R2* under purifying selection, no SNPs significantly associated with expression level could be found within the ± 5000 bp flanking regions of CPRs (Table 1, Additional files 4 and 5). In *CG17660*, detected as subject to purifying selection, and *CG14253* to balancing selection, although some SNPs in the non-CPR region were associated with expression level, these SNPs were not linked with SNPs in CPR (Additional files 4D and 5E). In *CHKov1*, detected as subject to positive selection, and *Cyp4d1* (cytochrome P450-4d1) and *Nmda1* (N-methyl-D-aspartate receptor- associated protein) to balancing selection, many SNPs in the coding and flanking regions were significantly associated with variation in expression level, and furthermore, these SNPs were linked with SNPs of CPRs (Table 1 and Additional files 4A, 5D and F). CPR of *CHKov1* was also linked with an insertion of a *Doc* transposable element. Thus, for these three genes, it is possible that natural selection operated on noncoding regions flanking CPRs, affecting variation in the expression level, and that these sequences were linked with CPRs.

**Fig. 3:**
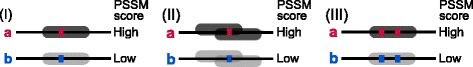
Patterns of estimated TFBS change caused by nucleotide changes in CPRs. **I** One TFBS change caused by one SNP. **II** Two or more TFBSs changes caused by changes at a single nucleotide site. **III** One TFBS change caused by nucleotide changes at two or more sites. Black lines indicate CPRs of each allele, *a* and *b*. Red and blue rectangles indicate SNP sites for each allele. Dark and light gray ellipses indicate acquired and lost TFBS, respectively

**Fig. 4:**
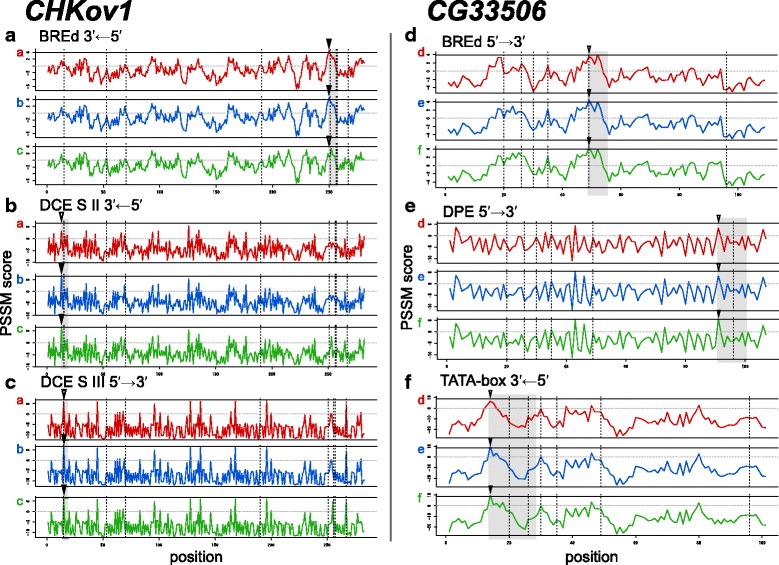
Distribution of PSSM scores for three different TFBSs along CPR sequences for *CHKov1* and *CG33506*. PSSM scores (log odds scoring matrix) for the binding sites of BREd (**a** for *CHKov1* and **d** for *CG33506*), DCE S II (**b** for *CHKov1*), DCE S III (**c** for *CHKov1*), DPE (**e** for *CG33506*), and TATA box (**f** for *CG33506*) at all positions in CPR sequences on the strand are shown. Gray dotted horizontal lines indicate a PSSM score of zero and black horizontal lines indicate PSSM scores at threshold values above which each transcription factor is likely to bind. Black dashed vertical lines indicate the position of SNPs found in the population. Black triangles indicate positions where PSSM scores for one or more alleles were larger than the threshold value (*closed triangle*), while other alleles had PSSM scores smaller than the threshold value (*open triangle*). Gray shading indicates the range of positions at which PSSM score are changed by mutations. Different colors (*red*, *blue*, *and green*) indicate differences in TFBS patterns caused by sequence variations. The color and alphabet (*a*-*f*) correspond with alleles shown in Fig. 2

**Fig. 5:**
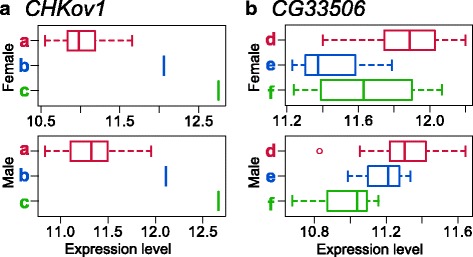
Expression level of different alleles of CPRs for *CHKov1* and *CG33506*. Expression levels of each allele for *CHKov1* (**a**) and *CG33506* (**b**) were retrieved from a database in female and male flies [16] found in a natural population Color (*red*, *blue*, *and gree*n) and alphabet (*a*-*f*) correspond to those in Fig. 2

CPRs of *CHKov1*, *CG11590*, *CG11660*, and *MBD*-*R2* were assigned as regulatory regions in which variant sequences caused the acquisition and/or loss of TFBS (Additional file 2), and likely increased in frequency through purifying selection or selective sweep (Fig. 2a and Additional files 3, 6, and 7). Almost all changes in TFBS were caused by one-nucleotide changes that affected the PSSM score (pattern I, Fig. 3). For CPRs of *CHKov1*, DCE (downstream core element) S II and III binding sites were lost for one of the derived alleles (a in Figs. 2a, 4b, and c). This loss resulted from a nucleotide change at only one SNP site in CPR of *CHKov1* from an ancestral to a derived allele (pattern II, Fig. 3). The BREd (downstream TFIIB recognition element) binding site was acquired for two derived alleles (a and b in Figs. 2a and 4a). Three SNP sites caused differences in PSSM scores within the BREd binding site (pattern III, Fig. 3). An ancestral allele (c in Fig. 2a) did not carry the BREd binding site. Among the three SNP sites, nucleotide mutations at the furthest downstream SNP site and at one of the remaining SNP sites were minimal requirements for acquiring the BREd binding site from the ancestral allele. In CPRs for *CHKov1*, differences in binding sites between ancestral and derived alleles were associated with variation in gene expression level that was subject to selection (Figs. 2a, 4a-c, and 5a).

CPRs for *brat*, *Nmda1*, *Cyp4d1*, and six unknown genes, namely *CG15743*, *CG9044*, *CG14253*, *CG6950*, *CG10463*, and *CG33506*, could be assigned as regulatory regions in which variant sequences caused changes in TFBSs (Additional file 2). Their differences in binding sites were associated with variation in gene expression levels and the sequence variations had been maintained through balancing selection (Figs. 2b, 4d-f and 5b and Additional files 8, 9 and 10). Almost all TFBS changes including those in *brat*, *Nmda1*, and *Cyp4d1* were caused by one nucleotide that affected the PSSM score (pattern I, Fig. 3). In CPR of the unknown gene *CG10463*, a nucleotide change at one SNP site for two alleles (a and b in Additional file 8I) was required to acquire the Inr binding site, whereas a nucleotide change at another SNP site abolished the binding site in allele a. Furthermore, there were four SNPs in the TATA box binding site. A nucleotide change at the furthest upstream SNP site decreased the PSSM score and those at other sites increased it. In CPR for an unknown gene *CG6950*, an allele (c in Additional file 8H) was inferred to have acquired the DCE S I binding site by nucleotide changes at three SNP sites (Additional file 9G). A nucleotide change in the furthest upstream region of these three SNP sites did not change any binding sites. The other two nucleotide changes were needed to acquire the DCE S I binding site. A nucleotide change at one SNP site could cause the acquisition of one TATA box binding site for allele b, and two SNPs in another binding site caused the loss of one TATA box binding site and the simultaneously acquisition of two TATA box binding sites for allele a (Additional file 9G). In CPR for the unknown gene *CG14253*, two nucleotide changes at two SNPs affected the motif ten element (MTE) binding site and its binding strength (Additional file 9E). One of the SNPs slightly changed the binding strength. Another SNP caused the loss of the binding site through a significant change of binding strength in allele e.

Variation in expression level may also be associated with copy number variation [29]. Our results could have been influenced by allele-specific duplication including a CPR rather than variation in a CPR. To identify copy number variation by allele-specific duplication, we analyzed read depth in each individual. Duplications were not detected in CPRs associated with differences in gene expression level and detected as being subject to selection. This result indicates that the changes of expression level we detected were not due to duplication.

## Discussion

We identified CPR sequence variations that had been subject to natural selection and associated them with differences in gene expression level in a natural population of *D. melanogaster*. Our results showed that nucleotide changes in CPR sequences caused variation in binding profiles, thereby affecting expression of regulated genes. Phylogenies of CPR sequences indicated that for several genes, variations in CPR sequences associated with changes in gene expression were maintained by balancing selection and that for several other genes, variant sequences of CPRs that changed gene expression levels had increased in frequency via selective sweep. Previous studies have shown that mutations in *cis*-regulatory regions underlie many phenotypic differences [2, 30, 31]. Recent whole-genome analyses have detected signals of selection in regulatory regions [8] using whole-genome sequences and ChIP-seq data from human populations and shown clear evidence for natural selection in binding sites of several transcription factors. Although previous studies could detect candidate sequence regions that had been subject to selection, they could not identify causal mutations responsible for variation in gene expression in natural populations. The present study has identified specific nucleotides in CPRs of *cis*-regulatory regions that could change binding profiles (as estimated by PSSM scores), thereby altering the expression levels of regulated genes, as candidate alleles for natural selection.

In the North Carolina population of *D. melanogaster*, the average nucleotide diversity (π) and Watterson’s θ_w_ of CPRs were similar to those of an entire genome and higher than those of CDS, although the sample sizes were unequal. These results indicated that although CPRs contained functional regions such as TFBS, these sequences were less conserved than those in coding sequences. Wasserman and Sandelin [31] and Bajic et al. [32, 33] showed that sequences in the regulatory regions of orthologous genes were much more divergent between mammalian species. The present study indicated that the sequences of CPRs were diverse even within a population, possibly providing a source for natural selection.

Nearly 80 % of expressed transcripts were significantly influenced by sex-biased gene expression. This finding suggests that sexual dimorphism for gene expression is a common pattern, in agreement with a previous report [16]. Only 2 % of expressed transcripts were significantly influenced by sequence-by-sex interaction and no enriched GO terms were detected for these transcripts. This finding indicates that CPRs are generally independent from sex-specific expression.

Two or more TFBS changes caused by one nucleotide and one TFBS change caused by two or more nucleotides were found in several CPRs. These TFBS changes would be required to alter expression levels that result in selective differences. For some genes, one nucleotide change in CPR resulted in gain and/or loss of two or more TFBS (pattern II, Fig. 3). Also, in one gene (*CG6950*), two nucleotide changes in CPR resulted in the loss of one TATA box as well as the acquisition of two different TATA boxes. In other words, one of the acquired TATA boxes moved to a different site as the result of the nucleotide changes. Although the nucleotide changes could cause significant changes in gene expression levels via several TFBS changes and moves, they may not have been sufficient to affect natural selection. In addition, for some genes, changes in expression levels associated with altered TFBS often required two to four nucleotide changes in CPRs. In this case, several different patterns of mutation could change the gene expression level via one TFBS change. These results indicate that only one major mutation in a CPR did not lead to significant changes in gene expression necessary for natural selection.

SNPs that could be detected as variations in control regions affecting differences in expression level could have been linked with neighboring sequences. In eight genes detected as being subject to natural selection, variation in expression levels was not explained by any SNP located in a flanking region of CPRs using association analysis (Additional files 4 and 5). In two genes, SNPs located in the region flanking CPR could explain the difference in expression level, and a test for linkage disequilibrium indicated that these SNPs were not linked with SNPs in CPR. Thus, SNPs within CPR and outside CPR regions may have independently affected changes in expression level. In *CHKov1*, *Cyp4d1*, and *Nmda1*, SNPs affecting expression level were located mostly in coding regions and were linked with those in CPR (Additional files 4A, 5D and F). Accordingly, for 10 of 13 genes, it could be concluded that natural selection has operated on the changes in expression level associated with variation in CPRs, whereas in three genes (*Cyp4d1*, *CHKov1* and *Nmda1*), the present analysis could not exclude the possibility that natural selection had operated on a flanking regulatory region linked with SNPs in CPRs (Table 1). In these three genes, the results could not determine whether SNPs in CPRs, coding regions, or non-coding regions flanking CPRs have been subject to natural selection for variation in expression level.

*CHKov1* encodes choline kinases and confers resistance to the sigma virus and organophosphate insecticides [34, 35]. Infection of the sigma virus and the effects of insecticide are associated with choline kinase activity. The sigma virus uses acetylcholine receptors to enter cells in *D. melanogaster* natural populations [36–38]. Organophosphate insecticides affect choline metabolism by inhibiting acetylcholine esterase activity [34]. In a European line of *D. melanogaster* with a different gene structure resulting from complex duplications and showing higher resistance, expression levels of *CHKov1* decreased, although not statistically significantly [35]. This finding suggested that lower expression levels of *CHKov1* decrease the production of proteins with the choline kinase domain and increase resistance to the sigma virus and insecticides. Our results indicated that in CPR of *CHKov1*, derived alleles with decreased expression levels had been subject to selective sweep. Thus, we inferred the following evolutionary scenario: (1) mutations at two or more nucleotide sites caused acquisition of the binding site BREd and/or a mutation at another nucleotide site caused the loss of binding sites DCE S II and DCE S III, (2) the alleles that acquired and/or lost the TFBSs have decreased in expression level in both sexes, and (3) the alleles increased in frequency through natural selection, resulting in increased viral resistance.

In previous studies, insertion of the *Doc* transposable element into the coding sequence of *CHKov1* was associated with increased resistance to the sigma virus [34, 35, 39]. Our results indicated the possibility of linkage between CPR and *Doc* insertion. Thus, nucleotide changes in CPR may be linked with *Doc* element insertion that affects the expression levels of *CHKov1*. However, it is possible that both nucleotide changes in CPR and *Doc* element insertion may increase viral and insecticidal resistance by reducing proteins expression with the choline kinase domain. *Doc* transposable element insertion induced expression of the *CHKov1* protein without a choline kinase domain [40]. The relationship between decreased expression levels of the choline kinase domain resulting from nucleotide changes in CPR and *Doc* element insertion is unclear. Both mutations may increase allele frequency in the population and resistance to virus.

Sequence variation in CPRs of *MBD*-*R2* and *CG17660* showed relatively little effect on gene expression, possibly because purifying selection may reduce the fitness difference between variants, and there was no relationship between gene expression and change in TFBSs. *CG11590* is involved in a biological process described as response to metal ion. It is unknown whether changes in its expression level affect its phenotype.

The present results suggest that CPRs of *brat*, *Nmda1*, *Cyp4d1*, *CG15743*, *CG9044*, *CG6950*, *CG14253*, *CG10463*, and *CG33506* were influenced by balancing selection for maintaining variation in gene expression levels. For these genes, different alleles with different expression levels resulted from TFBS with nucleotide differences. *Brat* and *CG9044* are involved in neuromuscular junction development and function [41, 42]. The neuromuscular junction terminals of *brat* mutants have reduced neurotransmission efficiency and defective endocytosis as a result of regulation of the bone morphogenetic protein (BMP) signaling pathway [41], although it is unknown how expression changes of the genes affect phenotype and fitness. Balancing selection may maintain diversity of neural and behavioral traits by variation in neuromuscular junctions. *Nmda1* encodes type 1 NMDA, which plays a role in the regulation of synaptic and behavioral plasticity and may be associated with olfactory learning, sleep, and long-term memory [43–45]. Behavioral polymorphism affected by CPR of *Nmda1* may be maintained by balancing selection, although it cannot be excluded that SNPs located in the coding sequences and flanking regions of CPRs may be important regions for selection.

Balancing selection may maintain a diversity of neural and behavioral traits by variation in neuromuscular junctions. In human populations, several regulatory regions have been shown to evolve under balancing selection [46–49]. In *D. melanogaster* populations, the 5′ flanking region of Dopa decarboxylase (*Ddc*), affecting longevity, has been suggested to maintain excess genetic variation through balancing selection [50]. However, these studies did not identify specific nucleotide sites under selection and did not show how different alleles in regulatory regions affected phenotype, gene expression, or interaction between DNA and proteins. The present study showed that two or three alleles for CPRs with different gene expression levels could be maintained by balancing selection. The functions of these genes are unknown, and thus, it is unclear why different levels of gene expression have been maintained by selection. Balancing selection for cosmopolitan inversions has been shown in *Drosophila* [51, 52]. Thus, in the detected CPRs, the allele frequencies may have been maintained by inversions rather than by balancing selection. The frequencies of three inversions (In(2 L)t, In(2R)NS and In(3R)Mo) are high in North America [53]. Two CPRs (*CG9044* and *CG14253*) were contained in the inversion of In(2L)t and In(3R)Mo and the other CPRs of the six genes inferred to have been maintained by balancing selection were not contained in these inversions [54]. Thus, the different sequences of CPRs of these six genes have been maintained by balancing selection for differences in gene expression but CPR variants of *CG9044* and *CG14253* may be maintained by inversion.

In this study, we could detect natural selection in a natural population for sequence variations in CPRs causing variations in expression level using genome and transcriptome data. This result indicates that natural variation in CPRs within a population is one of the sources of gene expression evolution. We cannot rule out factors other than expression difference as causes of natural selection, given that causal relationships among SNPs in CPRs, TFBS variants, and changes in expression level have not been demonstrated by experimental approaches. However, the substantial association among variants in CPR, TFBS, and expression level provided sufficient support for detecting candidate CPR variation that is subject to natural selection. Previous studies showed an association between expression variation and differences in binding strength of transcription factors in CPRs, and between sequence features and maximal transcription start activity [55, 56].

## Conclusions

We identified several genes with nucleotide changes in CPRs that resulted in altered gene expression levels through acquisition or loss of basal TFBSs in a natural population of *Drosophila melanogaster*. We also found that these nucleotide changes were increased in frequency by positive selection and were maintained by balancing selection. One of the positively selected genes may be associated with resistance to virus and insecticides. Some genes subject to balancing selection were associated with neuromuscular junction development and possibly plastic behavior and learning. The sequences of CPRs were diverse even within the population, possibly providing a source for natural selection.

## Methods

### Data sets

Sequence data for *D. melanogaster* were obtained from the DGRP [22], which contains fully sequenced inbred lines derived from a natural population in the Raleigh, North Carolina area. For CPRs mapped by genome-wide analysis [24], SNPs were extracted from the sequence data. Transcriptome data for each sex in the population were obtained from Ayroles et al. 2009 [16], who used a microarray of 14 perfect-match 25 bp oligonucleotides. Transcriptome expression was measured with the microarray using 3- to 5-day-old whole-body flies from the inbred lines. We used 29 inbred lines for which both genome sequences and transcriptome data were available. Because the SNPs data may include sequencing error, rare alleles with frequencies less than 1 % in the 168 inbred lines of DGRP were excluded. Not all SNPs were fixed within individual lines [22]. When one or more individual lines had heterozygous sites in CPR sequences, these lines were not used for CPR analysis. For the expressed transcript data, we used only transcripts in which the broad-sense heritability of expression level ranged from 0.3 to 1.0, indicating considerable genetic variation in gene expression [16]. We analyzed 11,454 CPRs in five major chromosome arms (2 L, 2R, 3 L, 3R, and X) and 10,096 expressed transcripts with heritability > 0.3. Some genes had more than one CPR identified by some transcripts and more than one expression level measured by some probe sets on the microarray, indicating variation caused by alternative splicing. Our data sets included all combinations of these CPRs and expression levels, in the expectation that CPR variation resulting from alternative splicing would show different expression levels of transcripts. We accordingly did not calculate mean expression levels of the same genes. Sites with undetermined nucleotides (denoted by “N”) were treated as follows: when one or more individual lines had undetermined nucleotides and others had the same fixed nucleotide at a site, the site was considered to be fixed for the nucleotide; when one or more individual lines had undetermined nucleotides and the rest of the individual lines showed polymorphic nucleotides, the lines with undetermined nucleotides were excluded from analysis. To avoid incorrect polymorphic patterns in transcripts in just a few individuals, we excluded transcripts if fewer than 20 individual lines shared transcripts without undetermined nucleotides. Excluding some of the lines and transcripts, we used 97.4 % of available transcripts.

Some transcripts were used to annotate CPR downstream of the transcript [24]. Given that new transcription start sites (TSSs) associated with 3′ untranslated regions (UTRs) are found in mammals [57], *Drosophila* may have similar start sites. However, Hoskins et al. (2011) concluded that TSSs in 3′ UTRs were unlikely to represent novel sites of transcription initiation and appeared to represent the 5′ ends of cytoplasmic transcript fragments, not independent promoters, in *Drosophila* [24, 58]. Considering that CPRs regulate expression level from upstream and not from downstream, sets of CPRs misannotated with 3′ UTR and upstream genes were removed and new sets of CPRs and downstream genes on the same strand were added.

### Analysis of associations between gene expression levels and polymorphisms in CPRs

A linear model was used to identify transcripts whose expression levels were changed by polymorphisms in CPRs. For each CPR, we used the following model: Y = μ + Seq + Sex + Seq × Sex + ε, where Y denotes the expression level of the gene transcript regulated by CPR, Seq the sequence at CPR, Sex the sex of the individual, Seq × Sex sequence-by-sex interaction, and ε the error variance. The sex term and the interaction with sequence were added to remove effects of sex-biased expression. Correction for multiple comparisons was performed using the Benjamini-Hochberg procedure [59] using a false discovery rate (FDR) of 5 %. These analyses were performed with R version 3.0.2. GO analysis was performed with DAVID Bioinformatics Resources [60].

### Detecting natural selection

To find potential regions evolving under directional or balancing selection in the natural population, we identified outlier regions using Tajima’s *D* test [28] for CPR sequences. Because Tajima’s *D* is influenced by demographic events, outlier regions from the observed Tajima’s *D* were determined using coalescent simulation using ms [61]. We modeled a feasible demographic history of the North Carolina population, which was inferred to have been generated from the admixture of African and European populations [27]. The demographic model assumed that the ancestral Africa population experienced a bottleneck event and that the European population was then colonized from the African population and underwent exponential growth. Finally, the admixture of African and European was assumed to generate the North Carolina population. Demographic parameter values were estimated by an approximate Bayesian computation approach [27, 62]. The sample size was assumed to be 29, which corresponds to the sample size of the data used. The length of the sequence was set to 160 bp, which corresponds to the median length of CPRs used in this study. The mutation and recombination rates were assumed to be 1.45 × 10^−9^ events/bp/generation [63] and 5.0 × 10^−7^ cM/bp [64], respectively. The simulations were performed 100,000 times to calculate Tajima’s *D* distribution as a null model to test neutrality of the observed values calculated from CPRs. A *P* value was then obtained from the proportion of simulation runs for which the value of Tajima’s *D* was greater than the observed values. *P* < 0.01 (by two-tailed test) was used as a criterion for purifying selection (and/or selective sweep) or balancing selection.

Evolutionary distances among sequences of CPRs identified as outliers by Tajima’s *D* were calculated using the maximum composite likelihood method. Neighbor-joining trees using CPR sequences were constructed with MEGA 5.2.2 [65]. Clade support was assessed by 1000 bootstrap replicates. An orthologous sequence from *D. simulans* obtained with BLAT [66] was used as an outgroup species.

### Binding-site analysis

For candidate CPRs influenced by natural selection, TFBSs were estimated using sequence motif analysis of both template and complementary strands included in the Biopython package [67], based on AlignACE [68] and MEME [69]. This method approximates functionality with a unified motif object implementation. We used a PSSM, where the log odds of finding a motif against the background in which A, C, G, and T are equally likely and its balanced threshold approximately satisfies some relationship between the false-positive and -negative rate. The threshold of the false-negative rate/false-positive rate was 1000. Thirteen known DNA patterns linked to RNA polymerase II core promoters in the JASPAR3 POLII database [25] were used for estimation. Although DNA with these patterns do not necessarily bind to a specified protein, we called the patterns TFBSs for convenience. To search for a substantial number of SNPs or combinations associated with acquisition and loss of TFBSs, PSSM scores along with artificial sequences having each mutation in the TFBS and its combinations were calculated.

### Linkage disequilibrium and association analysis

To test for linkage between CPRs and neighboring regions, haplotype blocks were identified with Haploview [70]. We obtained haplotype blocks within which linkage disequilibrium occurs. A haplotype block was defined as a region within which there is little evidence for historical recombination [71]. The haplotype blocks were identified by evaluation of pairwise linkage disequilibrium between SNPs within ± 5000 bp of flanking regions of CPRs. To investigate whether expression levels were explained by SNPs in neighboring regions rather than those in CPRs, we tested for association between expression levels and SNPs within ± 5000 bp from CPRs using the Wald test. Correction for multiple comparisons was performed using the Benjamini-Hochberg procedure [59] with a false discovery rate (FDR) of 1 %. These analyses were performed with PLINK 1.07 [72].

### Duplication analysis

Differences in expression levels explained by changes in sequences may be influenced by allele-specific duplications that include a CPR. To identify copy number variation by duplication, we analyzed read depth in Illumina genome sequences. We removed 3′ end regions in which Illumina quality scores were less than 10. We also excluded from the analysis reads in which quality scores were less than 20 for 80 % or more of sites. The reads were mapped with BWA 0.7.12 [73] and the depth of coverage was calculated with SAMtools 1.2 [74]. Allele-specific duplications including a CPR were considered to have occurred when read depths of CPRs with ± 200-bp flanking regions were twice as great as the average for whole genes on the same chromosome.

## Additional files

Additional file 1:
**Table List of genes for which CPRs were analyzed.** (XLSX 791 kb) Additional file 2:
**Table List of acquired and/or lost TFBS within CPR detected natural selection.** (XLSX 51 kb) Additional file 3:
**Phylogeny of CPR for which sequence variation could explain gene expression variation and was subject to purifying selection or selective sweep.** Neighbor-joining trees for different alleles (a-g) of CPR are drawn for *MBD*-*R2* (A), *CG11590* (B), and *CG17660* (C). *Drosophila simulans* was used as an outgroup (o). Bootstrap values are shown for nodes with greater than 60 % support. (PDF 38 kb) Additional file 4:
**Estimated regions of linkage disequilibrium and associations between SNPs and expression levels in regions flanking CPRs for which sequence variation could explain gene expression variation and was subject to purifying selection or selective sweep.** The flanking regions (±5000 bp) of CPRs for *CHKov1* (A), *MBD*-*R2* (B), *CG11590* (C), and *CG17660* (D) are shown. Gray shading indicates haplotype blocks within which linkage disequilibrium could be found. Orange bars indicate coding region. Green bar indicates CPR. Each dot indicates false discovery rate (FDR) using the Wald test for association between expression level and SNPs. Horizontal line indicates FDR threshold (α = 0.01). (PDF 215 kb) Additional file 5:
**Estimated regions of linkage disequilibrium and associations between SNPs and expression level in regions flanking CPRs for which sequence variation could explain gene expression variation and was subject to purifying selection or selective sweep.** Flanking regions (±5000 bp) of CPRs for *CG15743* (A), *CG9044* (B), *brat* (C), *Cyp4d1* (D), *CG14253* (E), *Nmda1* (F), *CG6950* (G), *CG10463* (H), and *CG33506* (I) are shown. Gray shades indicate haplotype blocks within which linkage disequilibrium could be found. Orange bars indicate coding region. Green bar indicates CPR. Each dot indicates a false discovery rate value (FDR) using the Wald test for the association between expression levels and SNPs. Horizontal line indicates FDR threshold (α = 0.01). (PDF 626 kb) Additional file 6:
**Distribution of PSSM scores along CPR sequences for which sequence variation could explain gene expression variation and was subject to purifying selection or selective sweep.** PSSM scores (log odds finding motifs) for the binding sites of BREd (A and B), DCE S II (C), DCE S III (C), and MTE (C) at all positions along CPR sequences on the strand are shown. Gray dotted horizontal lines indicate a PSSM score of zero and black horizontal lines indicate PSSM scores at threshold values above which each transcription factor is likely to bind. Black dashed vertical lines indicate the position of SNPs found in the population. Black triangles indicate positions where PSSM scores for one or more alleles were higher than the threshold value (closed triangle), while other alleles had PSSM scores lower than the threshold value (open triangle). Gray shading indicates the range of positions at which mutations affected the altered PSSM score. Different colors (red, blue, and green) indicate differences in TFBS patterns caused by sequence variation. The color and alphabet (a-g) correspond to those in Additional file 3. (PDF 103 kb) Additional file 7:
**Expression levels of different alleles of CPRs for which sequence variation could explain gene expression variation and was subject to purifying selection or selective sweep.** Expression levels of each allele using microarrays in female and male flies [16] found in a natural population for *MBD*-*R2* (A), *CG11590* (B), and *CG17660* (C) are from the database. Color (red, blue, and green) and alphabet (a-g) correspond to those in Additional file 3. (PDF 41 kb) Additional file 8:
**Phylogeny of CPRs for which sequence variation could explain gene expression variation and was subject to balancing selection.** Neighbor-joining trees for different alleles (a-e) of CPR are drawn for *CG15743* (A), *CG9044* (B), *brat* (C), *Cyp4d1* (D), *CG14253* (E), *Nmda1* (F), *CG6950* (G), and *CG10463* (H). *Drosophila simulans* was used as an outgroup (o). Bootstrap values are shown for nodes with greater than 60 % support. (PDF 46 kb) Additional file 9:
**Distribution of PSSM score along CPR sequences for which sequence variation could explain gene expression variation and was subject to balancing selection.** PSSM scores (log odds finding motifs) for the binding sites of DCE S II (A and F), TATA box (A, C, G, and H), Inr (B, D, and H), MTE (E), BREu (G), DPE (G), and DCE S I (G), at all positions along CPR sequences on the strand are shown. Gray dotted horizontal lines indicate a PSSM score of zero and black horizontal lines indicate PSSM scores at threshold values above which each transcription factor is likely to bind. Black dashed vertical lines indicate the position of SNPs found in the population. Black triangles indicate positions where PSSM scores for one or more alleles were higher than the threshold value (closed triangle), while other alleles had PSSM scores lower than the threshold value (open triangle). Gray shading indicates the range of positions at which mutations affected the altered PSSM score. Different colors (red, blue, green, and yellow) indicate differences in TFBS patterns caused by sequence variation. The color and alphabet (a-e) correspond to those in Additional file 8. (PDF 200 kb) Additional file 10:
**Expression level of different alleles of CPRs for which sequence variations could explain gene expression variations and were subject to balancing selection.** Expression levels of each allele were from the database using microarrays in female and male flies [16] found in a natural population for *CG15743* (A), *CG9044* (B), *brat* (C), *Cyp4d1* (D), *CG14253* (E), *Nmda1* (F), *CG6950* (G) and *CG10463* (H). Color (red, blue, green, and yellow) and alphabet (a-e) correspond to those in Additional file 8. (PDF 53 kb)

## Abbreviations

CPR: core promoter region
PSSM: position-specific scoring matrix
TFBS: transcription factor binding site
TSS: transcription start site
