# Supplementary figures and images for: Natural selection in a population of Drosophila melanogaster explained by changes in gene expression caused by sequence variation in core promoter regions
Source: BMC Evol Biol. 2016 Feb 9;16:35. doi: 10.1186/s12862-016-0606-3 (PMC4748610; doi:10.1186/s12862-016-0606-3)

**A** *MBD-R2*

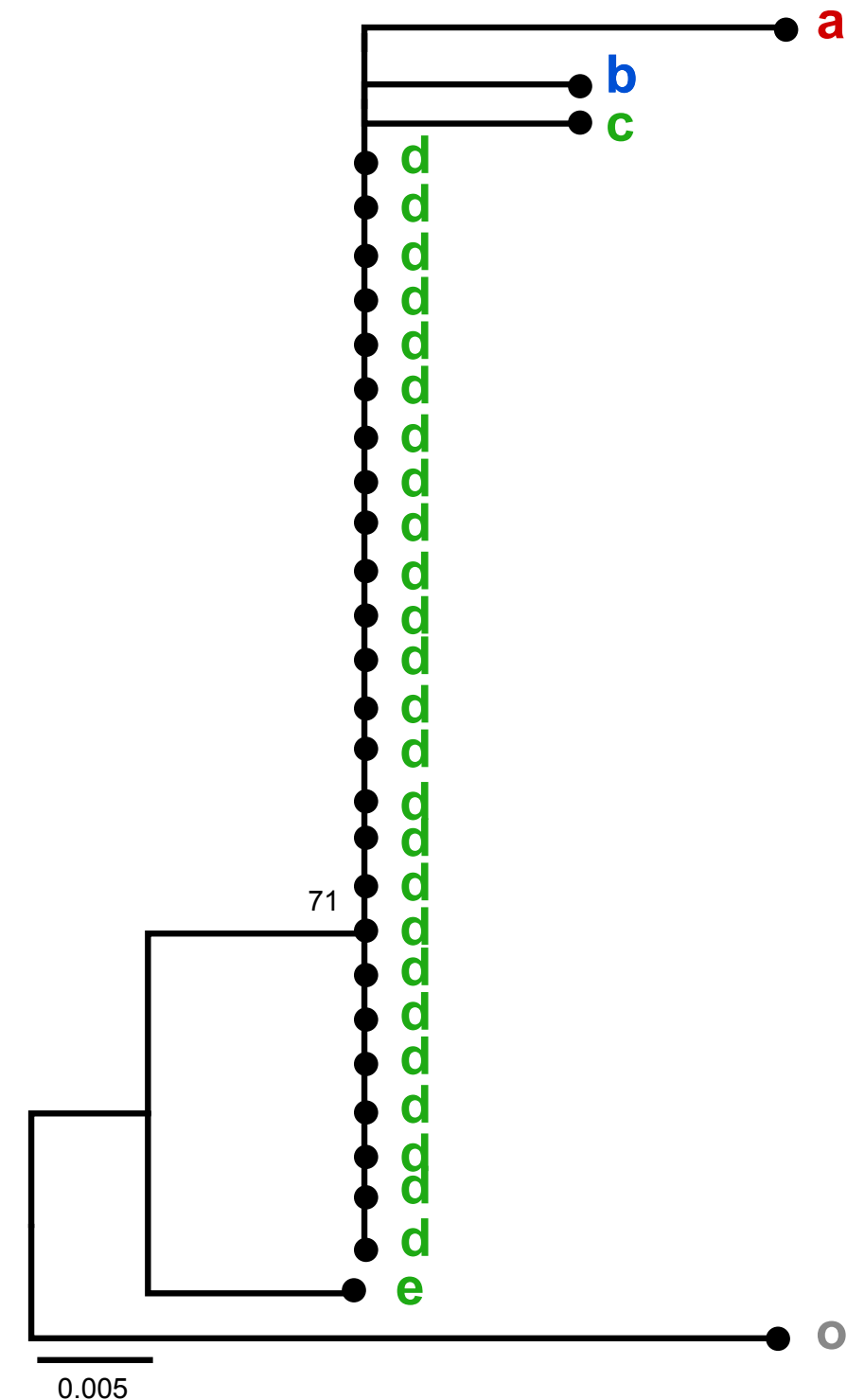

**B** *CG11590*

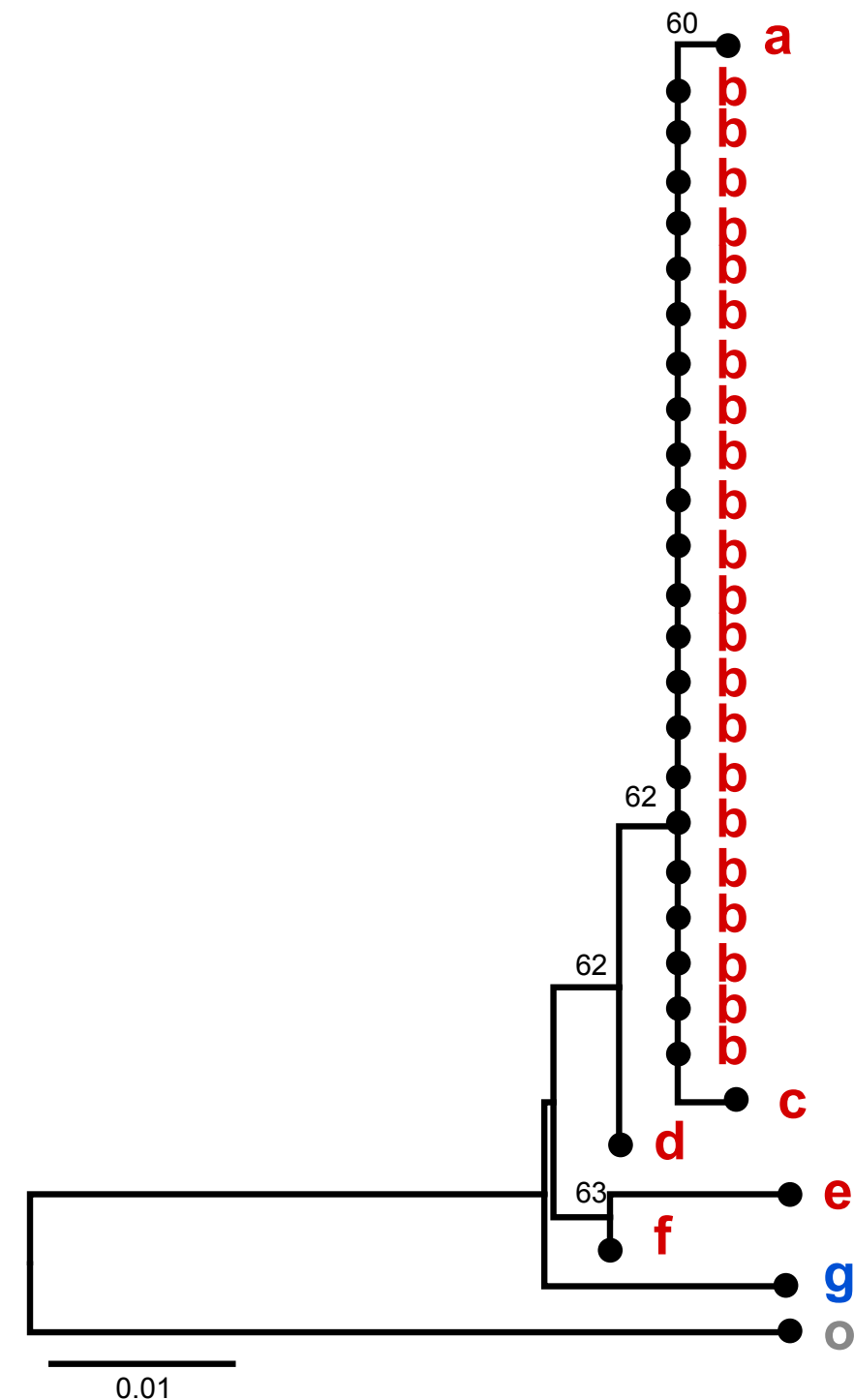

**C** *CG17660*

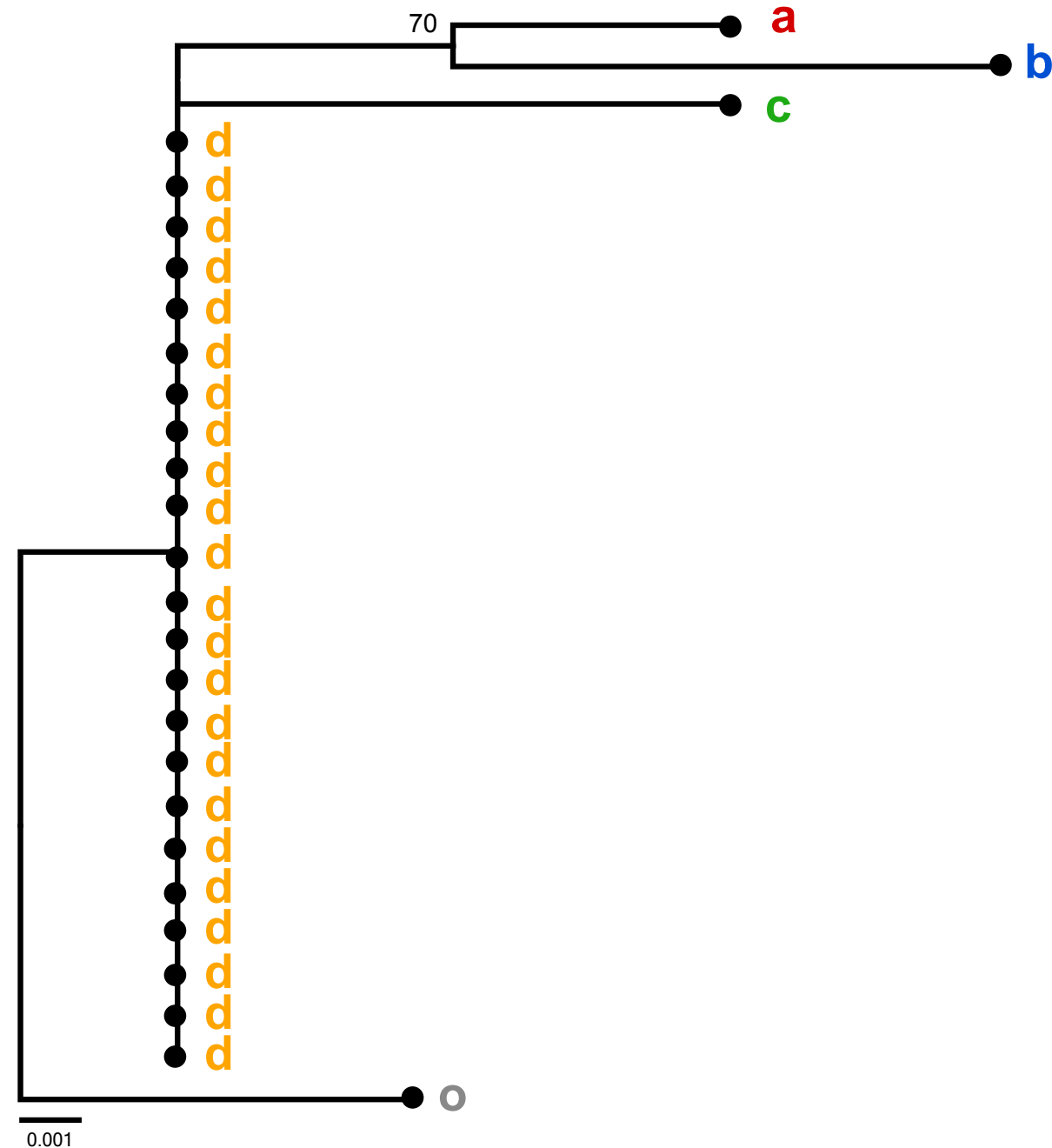

Supplement: Additional file 3: — Phylogeny of CPR for which sequence variation could explain gene expression variation and was subject to purifying selection or selective sweep. Neighbor-joining trees for different alleles (a-g) of CPR are drawn for MBD-R2 (A), CG11590 (B), and CG17660 (C). Drosophila simulans was used as an outgroup (o). Bootstrap values are shown for nodes with greater than 60 % support. (PDF 38 kb) [file 12862_2016_606_MOESM3_ESM.pdf]

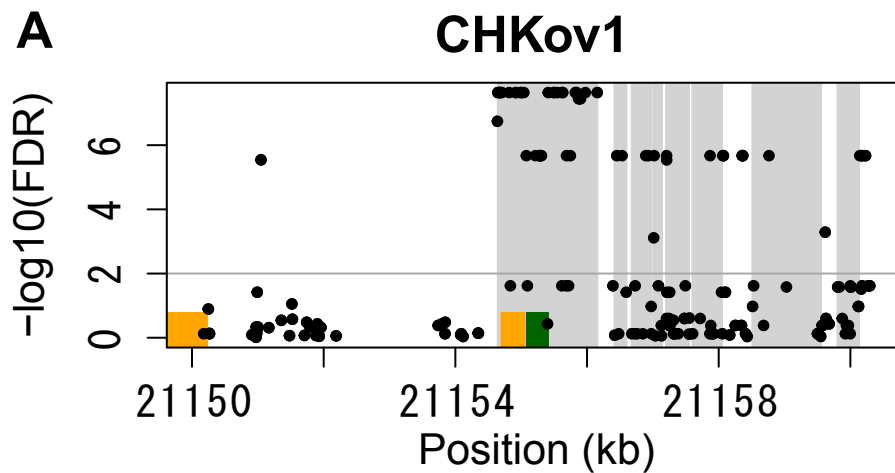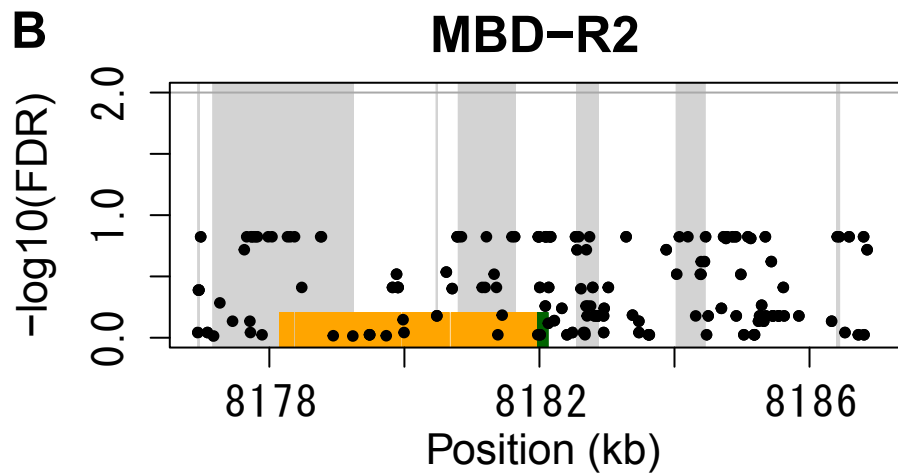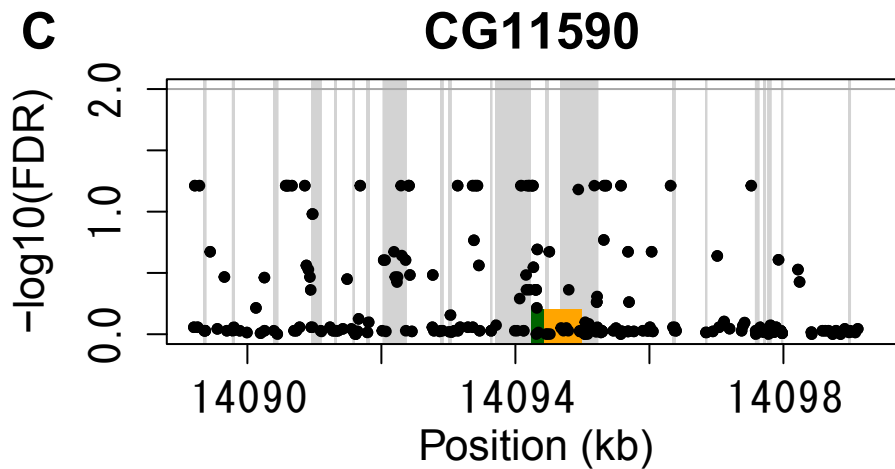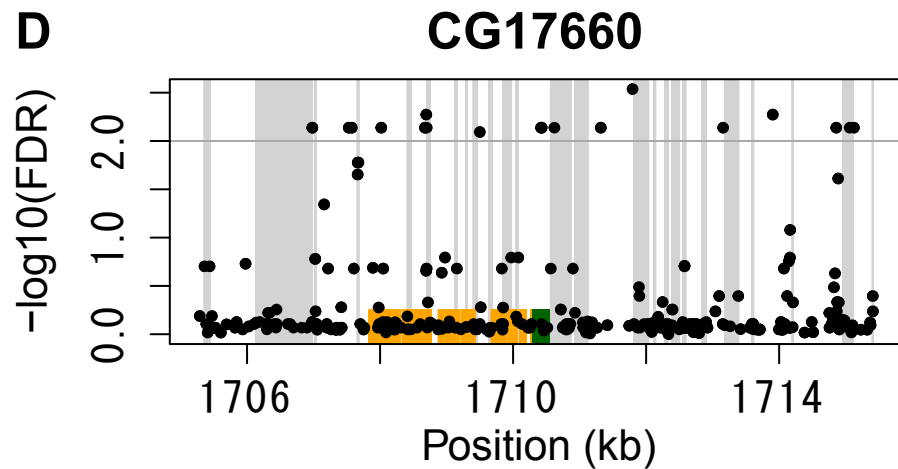

Supplement: Additional file 4: — Estimated regions of linkage disequilibrium and associations between SNPs and expression levels in regions flanking CPRs for which sequence variation could explain gene expression variation and was subject to purifying selection or selective sweep. The flanking regions (±5000 bp) of CPRs for CHKov1 (A), MBD-R2 (B), CG11590 (C), and CG17660 (D) are shown. Gray shading indicates haplotype blocks within which linkage disequilibrium could be found. Orange bars indicate coding region. Green bar indicates CPR. Each dot indicates false discovery rate (FDR) using the Wald test for association between expression level and SNPs. Horizontal line indicates FDR threshold (α = 0.01). (PDF 215 kb) [file 12862_2016_606_MOESM4_ESM.pdf]

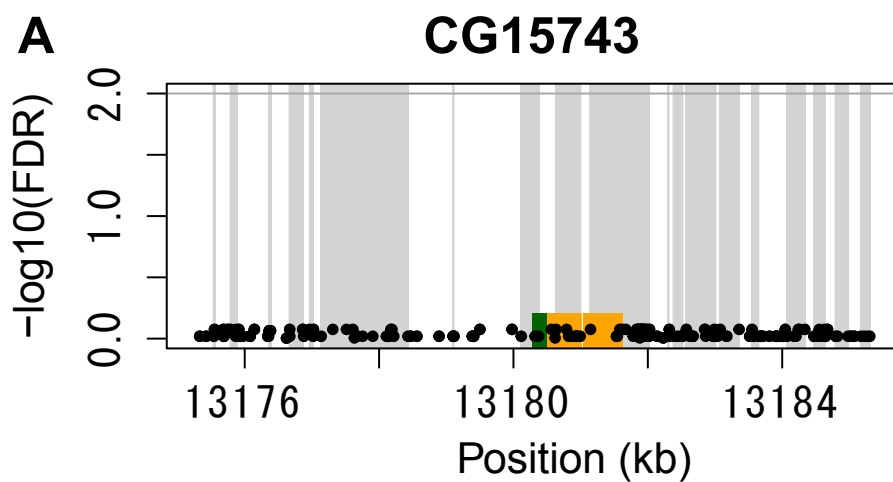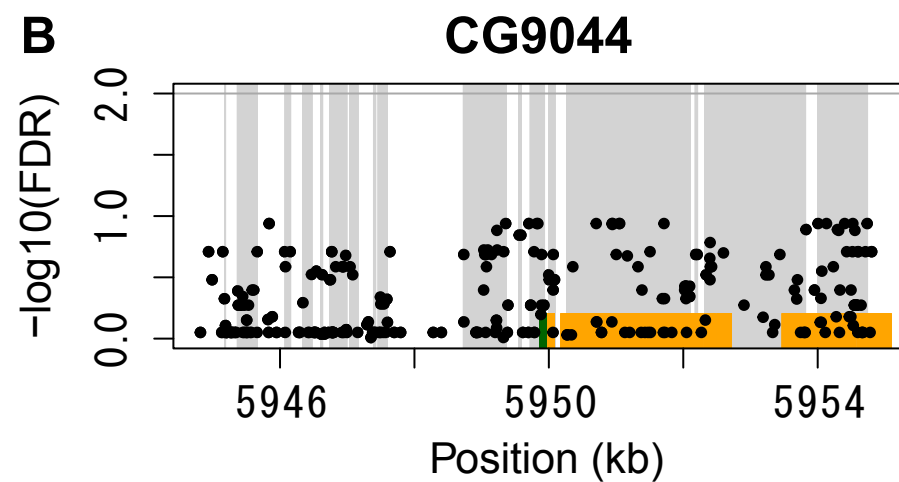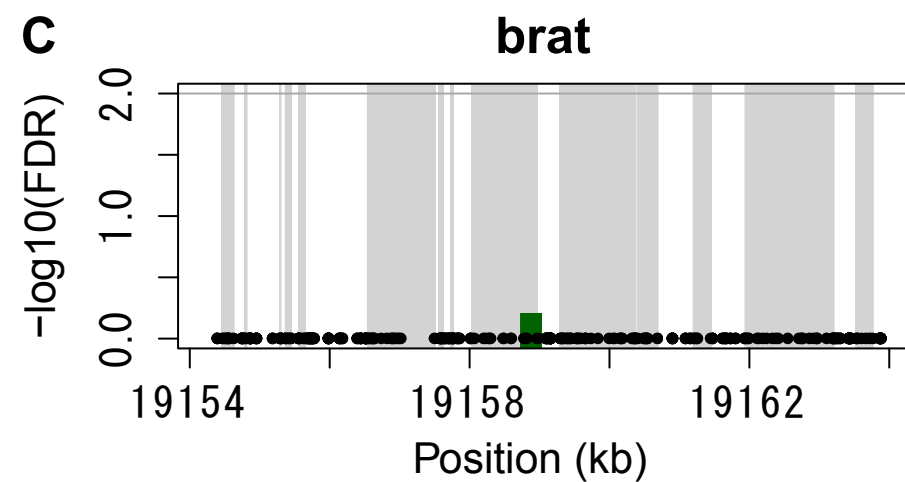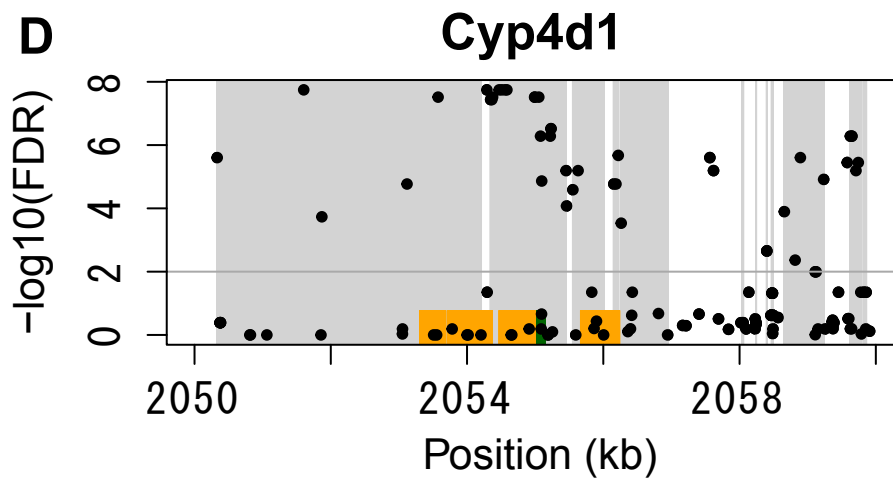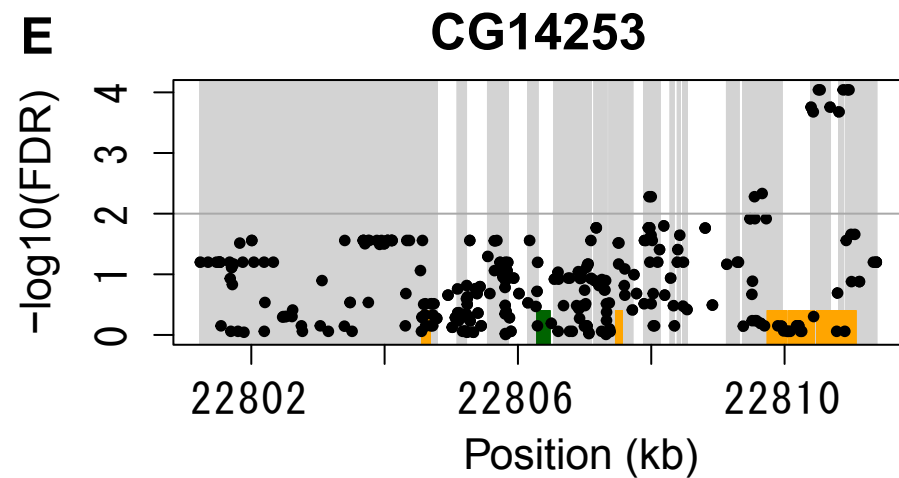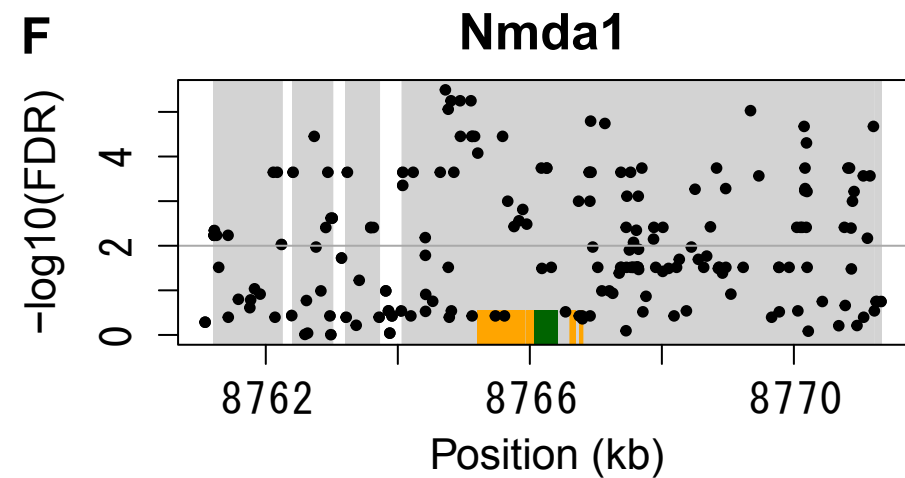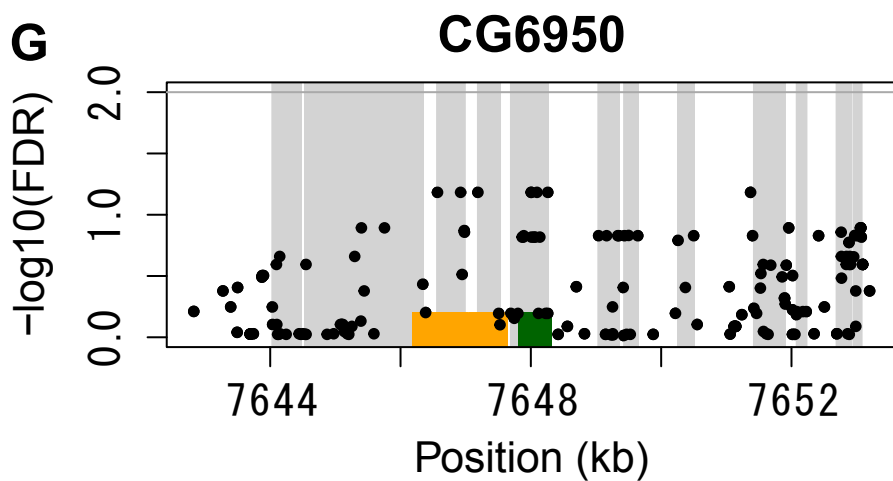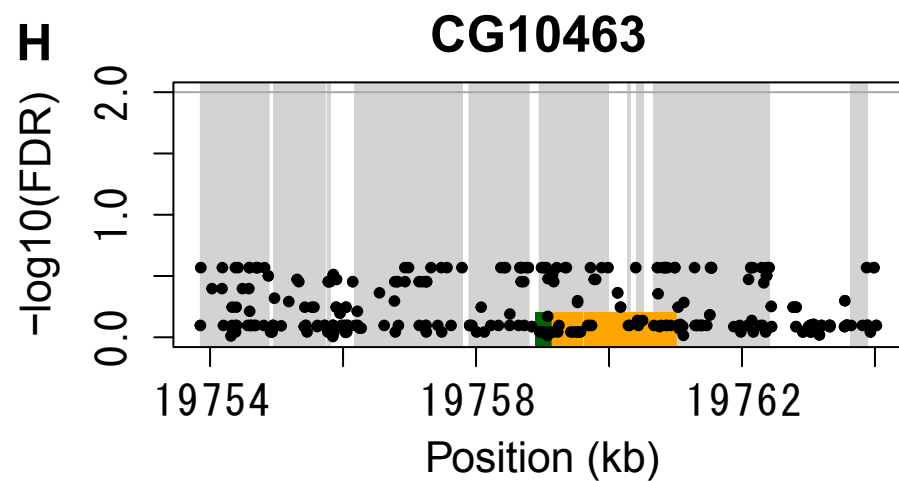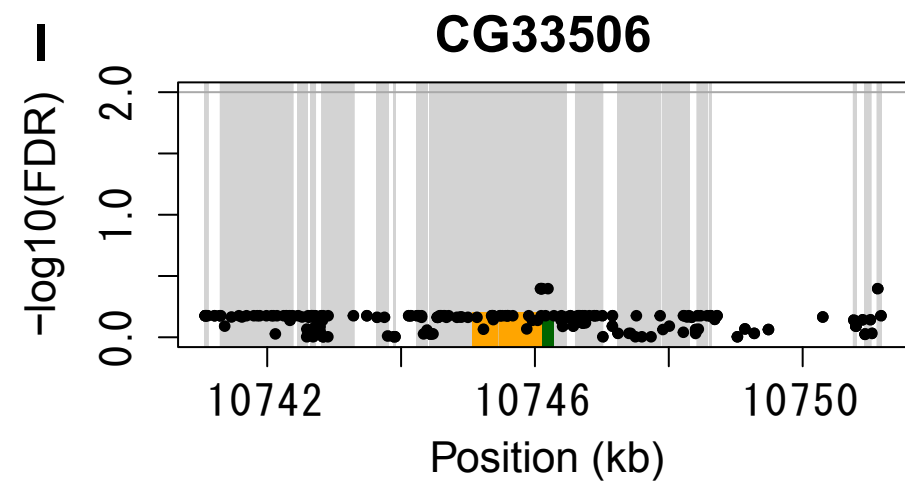

Supplement: Additional file 5: — Estimated regions of linkage disequilibrium and associations between SNPs and expression level in regions flanking CPRs for which sequence variation could explain gene expression variation and was subject to purifying selection or selective sweep. Flanking regions (±5000 bp) of CPRs for CG15743 (A), CG9044 (B), brat (C), Cyp4d1 (D), CG14253 (E), Nmda1 (F), CG6950 (G), CG10463 (H), and CG33506 (I) are shown. Gray shades indicate haplotype blocks within which linkage disequilibrium could be found. Orange bars indicate coding region. Green bar indicates CPR. Each dot indicates a false discovery rate value (FDR) using the Wald test for the association between expression levels and SNPs. Horizontal line indicates FDR threshold (α = 0.01). (PDF 626 kb) [file 12862_2016_606_MOESM5_ESM.pdf]

# A MBD-R2

BREd 3'←5'

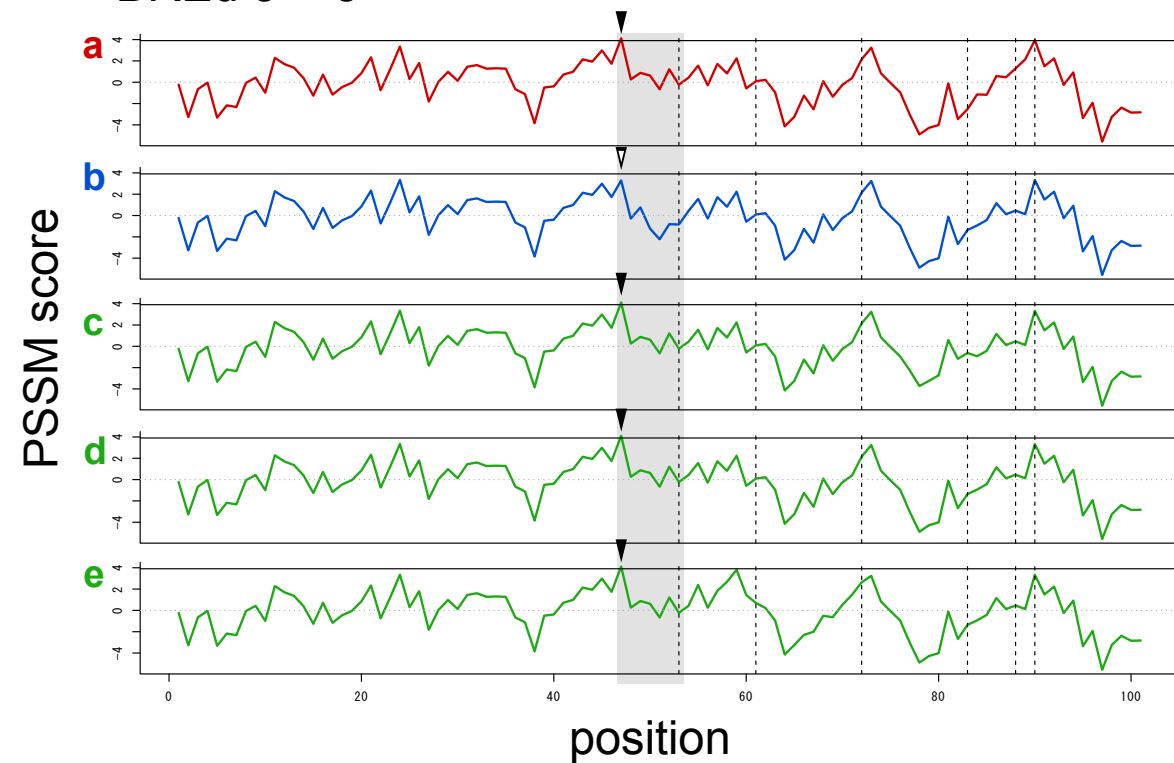

# B CG11590

BREd 5'→3'

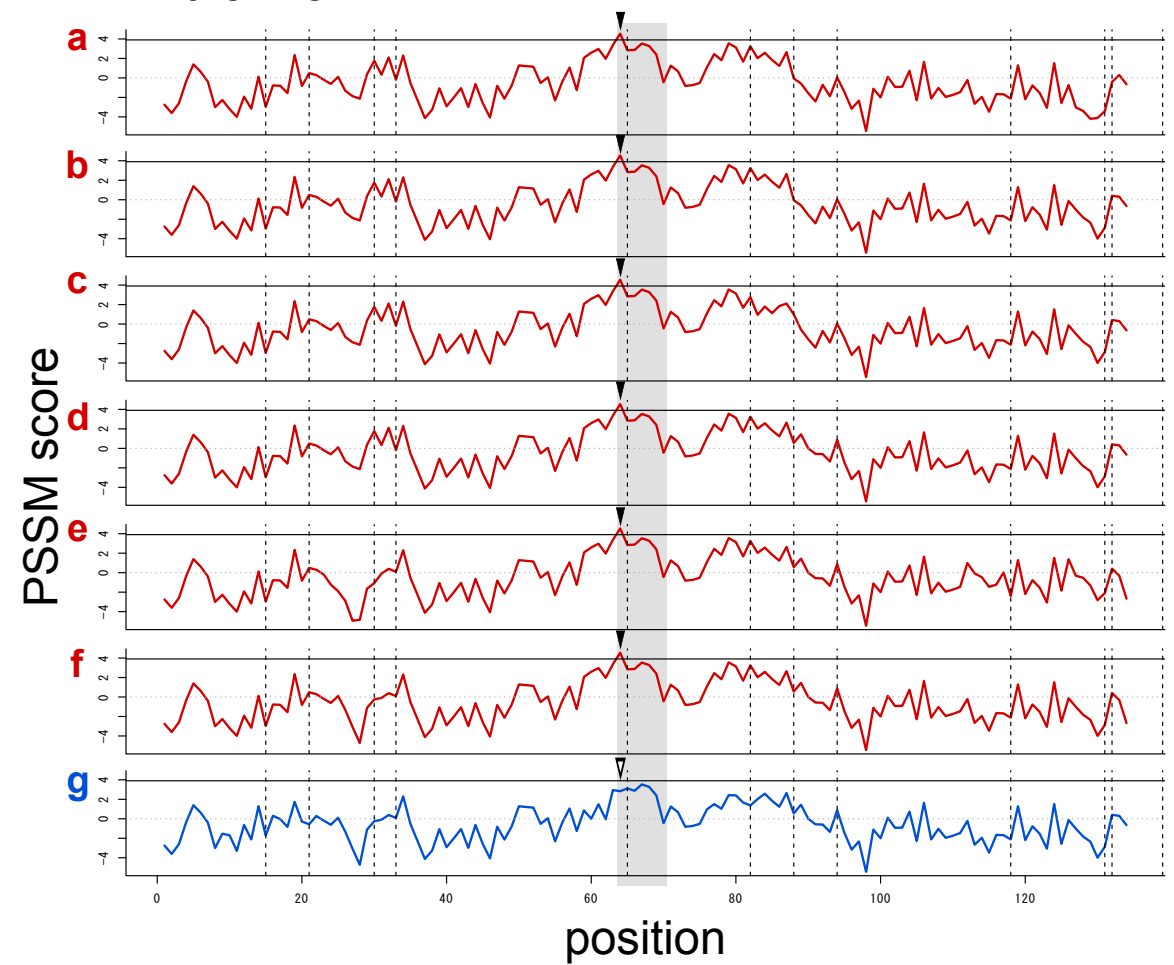

# C CG17660

DCE S II 5'→3'

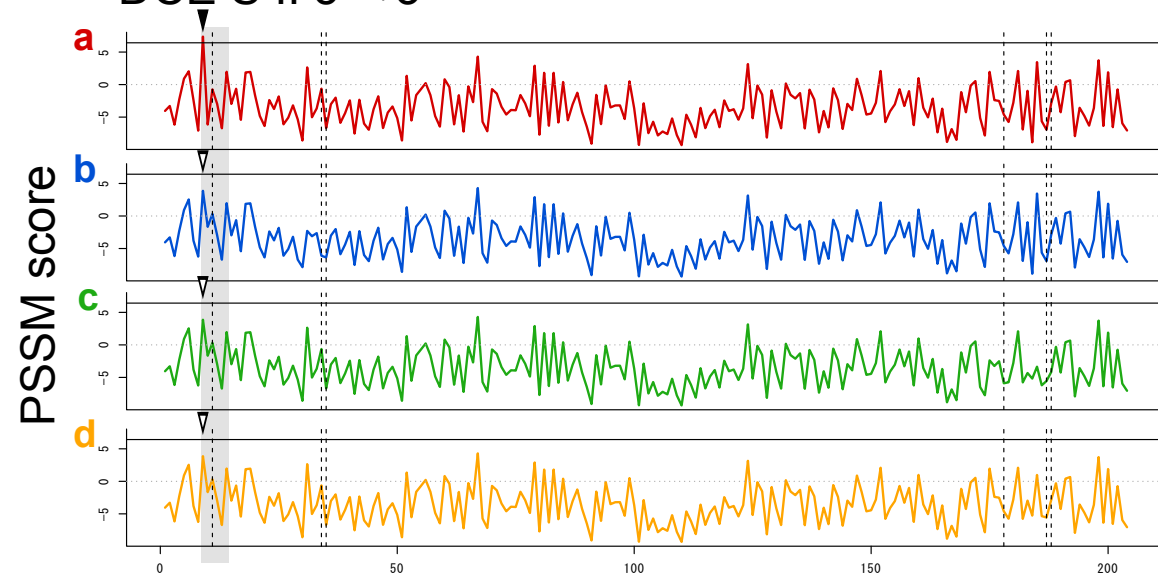

DCE S III 3'←5'

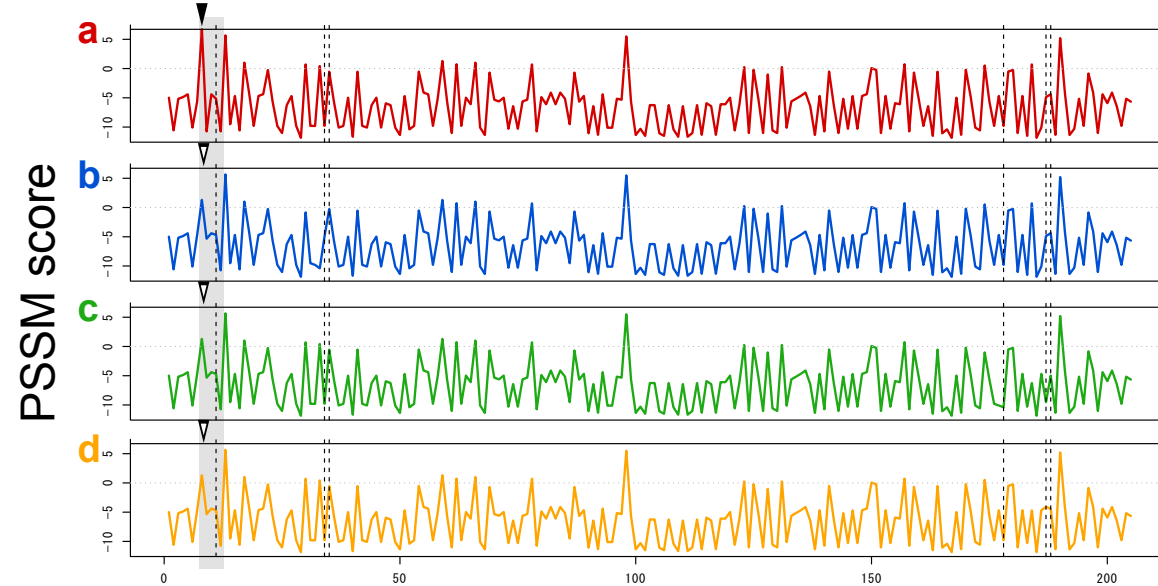

MTE 5'→3'

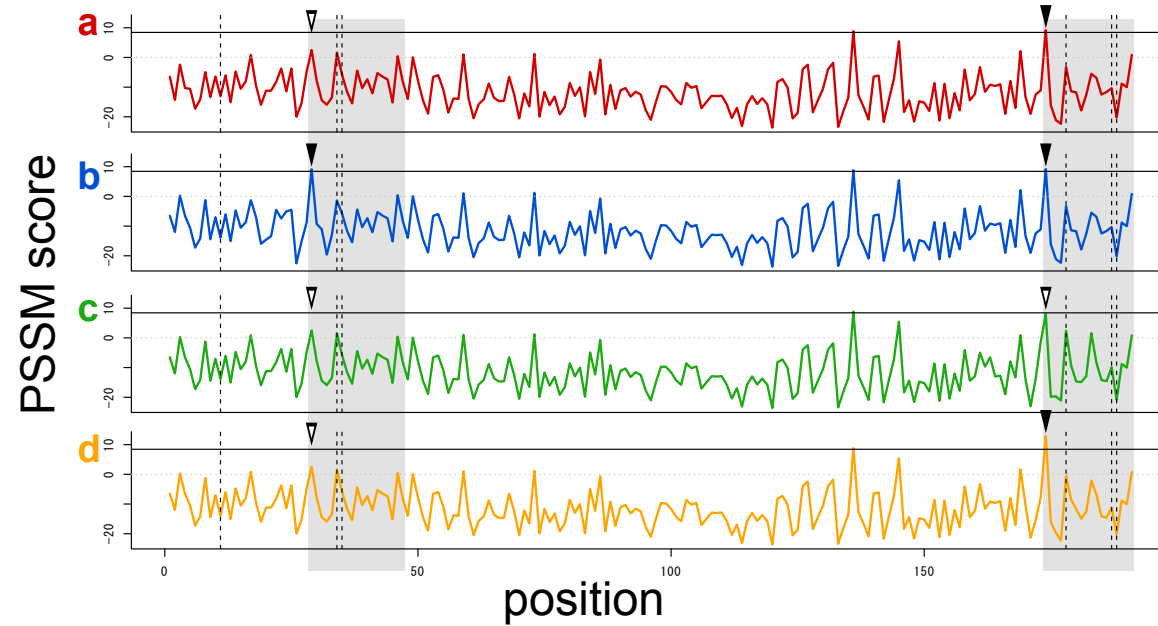

Supplement: Additional file 6: — Distribution of PSSM scores along CPR sequences for which sequence variation could explain gene expression variation and was subject to purifying selection or selective sweep. PSSM scores (log odds finding motifs) for the binding sites of BREd (A and B), DCE S II (C), DCE S III (C), and MTE (C) at all positions along CPR sequences on the strand are shown. Gray dotted horizontal lines indicate a PSSM score of zero and black horizontal lines indicate PSSM scores at threshold values above which each transcription factor is likely to bind. Black dashed vertical lines indicate the position of SNPs found in the population. Black triangles indicate positions where PSSM scores for one or more alleles were higher than the threshold value (closed triangle), while other alleles had PSSM scores lower than the threshold value (open triangle). Gray shading indicates the range of positions at which mutations affected the altered PSSM score. Different colors (red, blue, and green) indicate differences in TFBS patterns caused by sequence variation. The color and alphabet (a-g) correspond to those in Additional file 3. (PDF 103 kb) [file 12862_2016_606_MOESM6_ESM.pdf]

# A *MBD-R2*

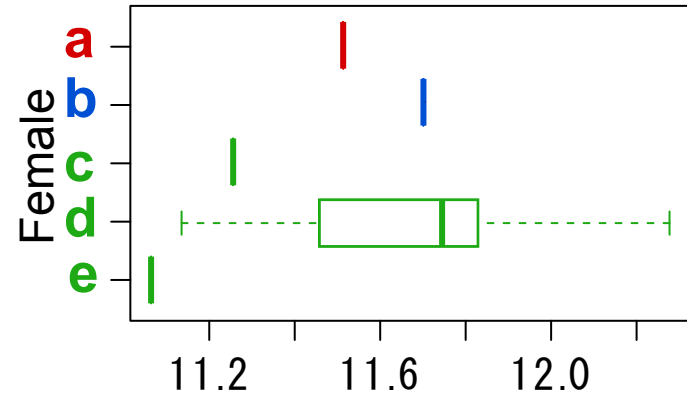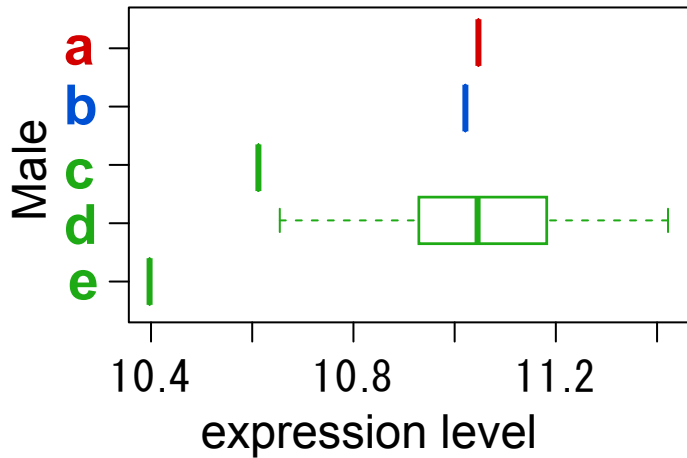

# B *CG11590*

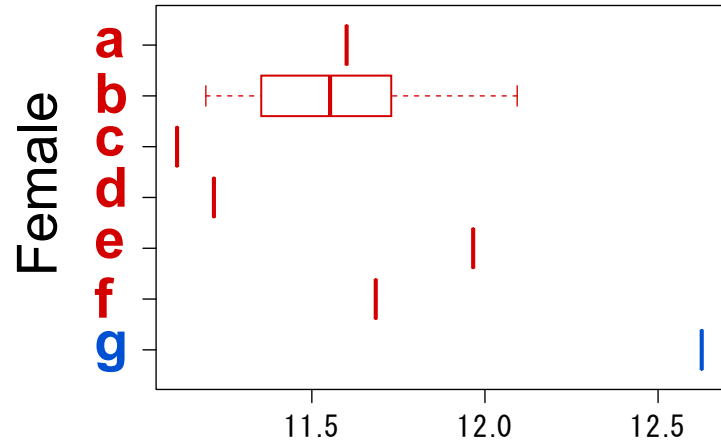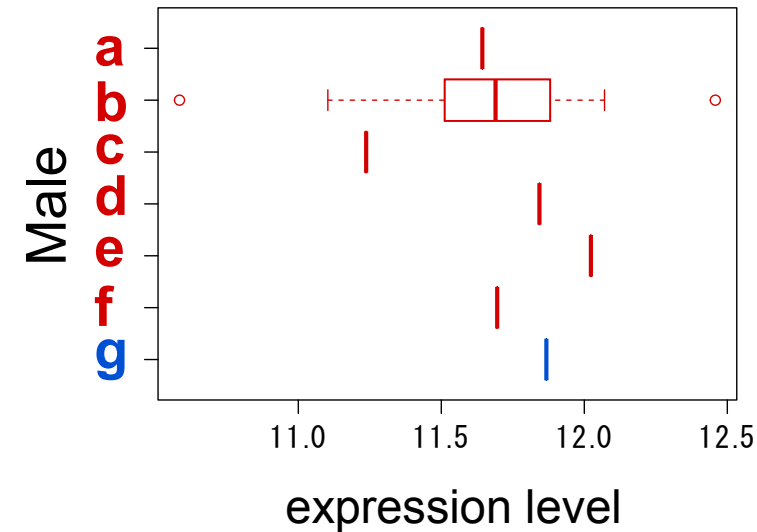

# C *CG17660*

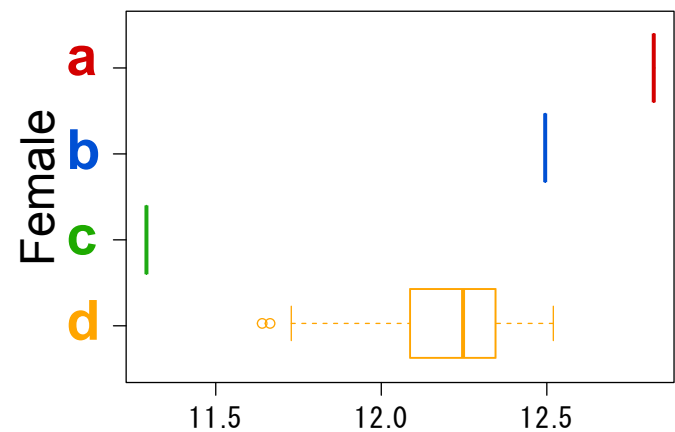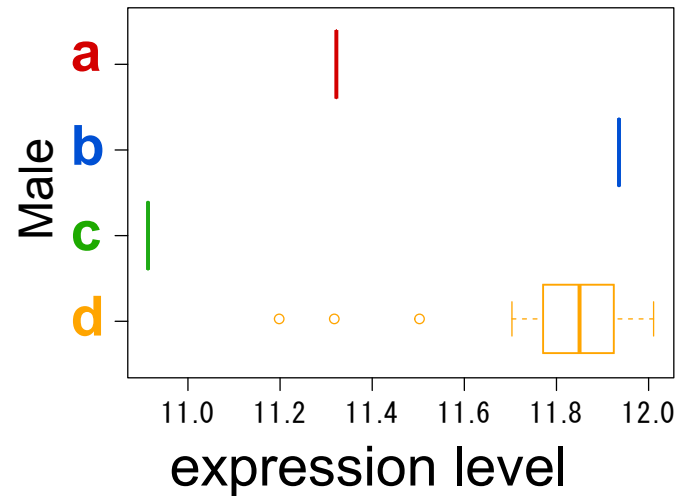

Supplement: Additional file 7: — Expression levels of different alleles of CPRs for which sequence variation could explain gene expression variation and was subject to purifying selection or selective sweep. Expression levels of each allele using microarrays in female and male flies [16] found in a natural population for MBD-R2 (A), CG11590 (B), and CG17660 (C) are from the database. Color (red, blue, and green) and alphabet (a-g) correspond to those in Additional file 3. (PDF 41 kb) [file 12862_2016_606_MOESM7_ESM.pdf]

**A CG15743**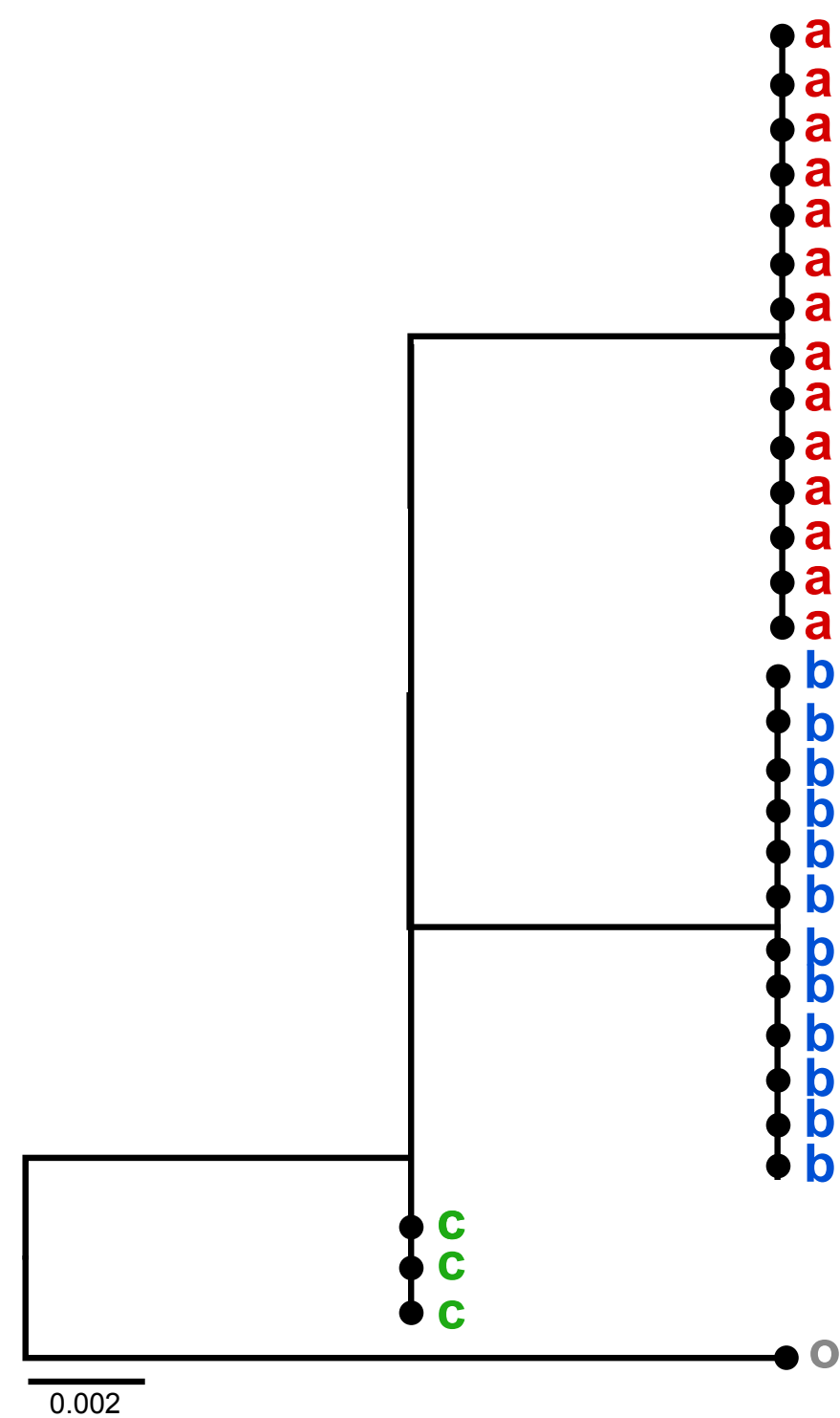**B CG9044**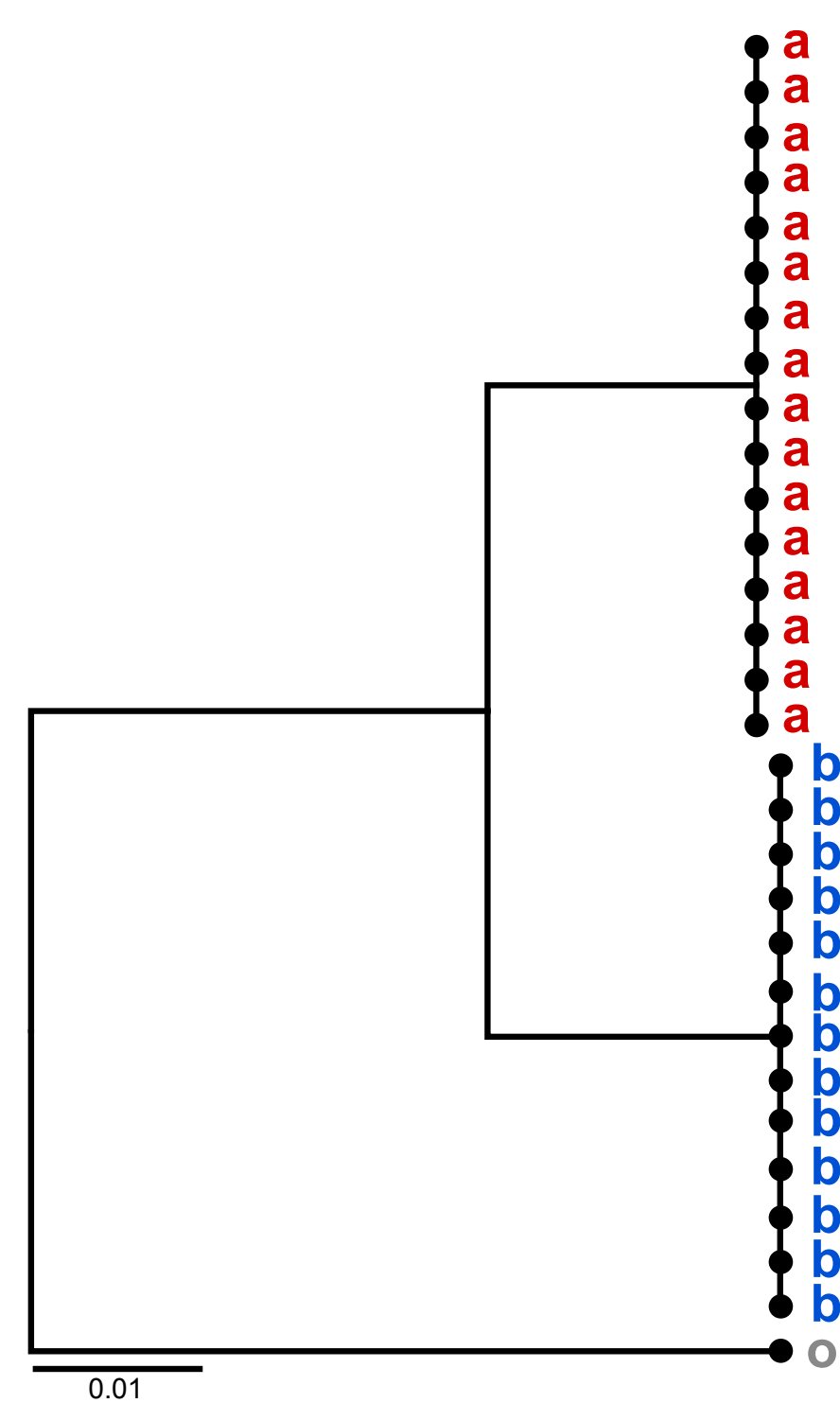**C brat**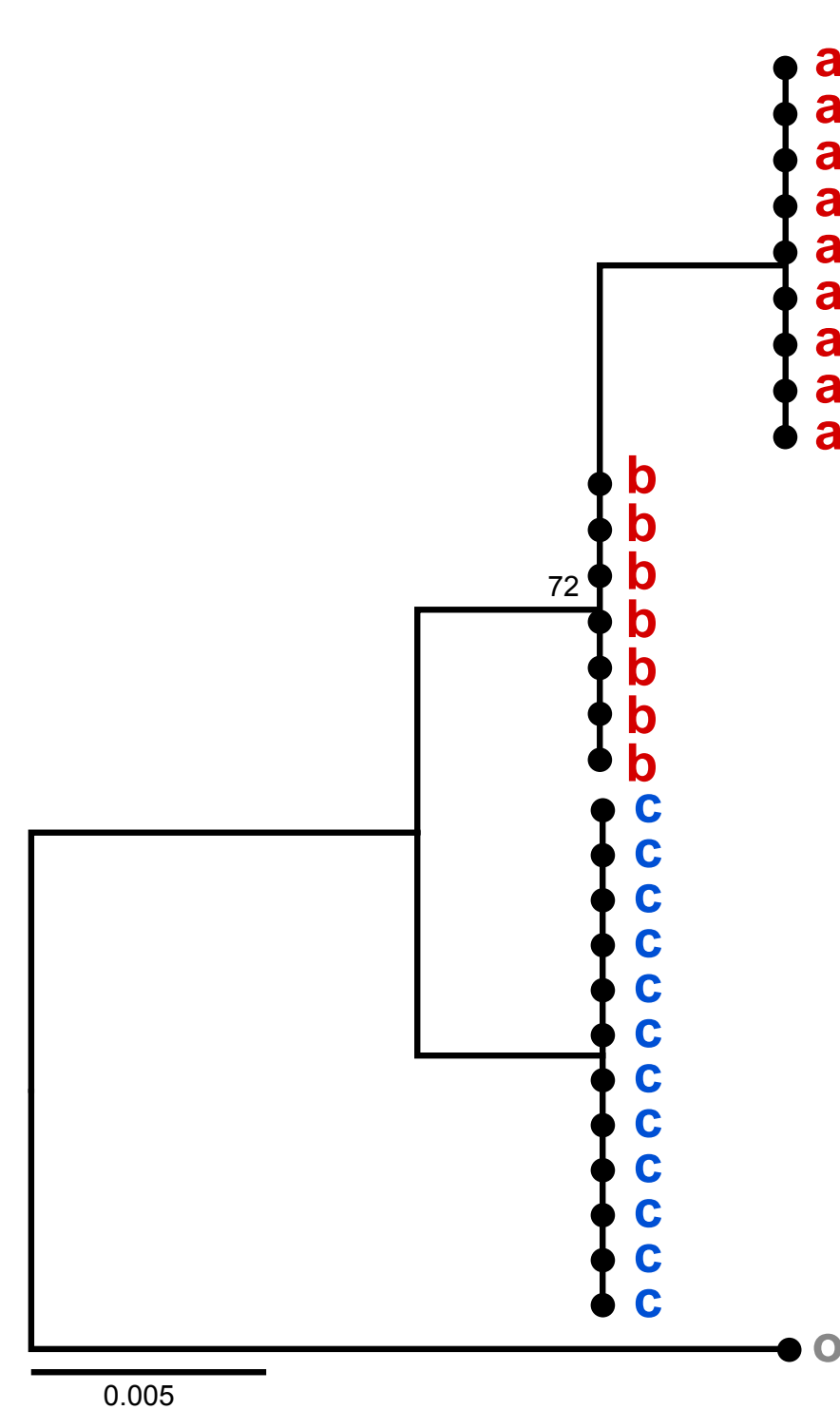**D Cyp4d1**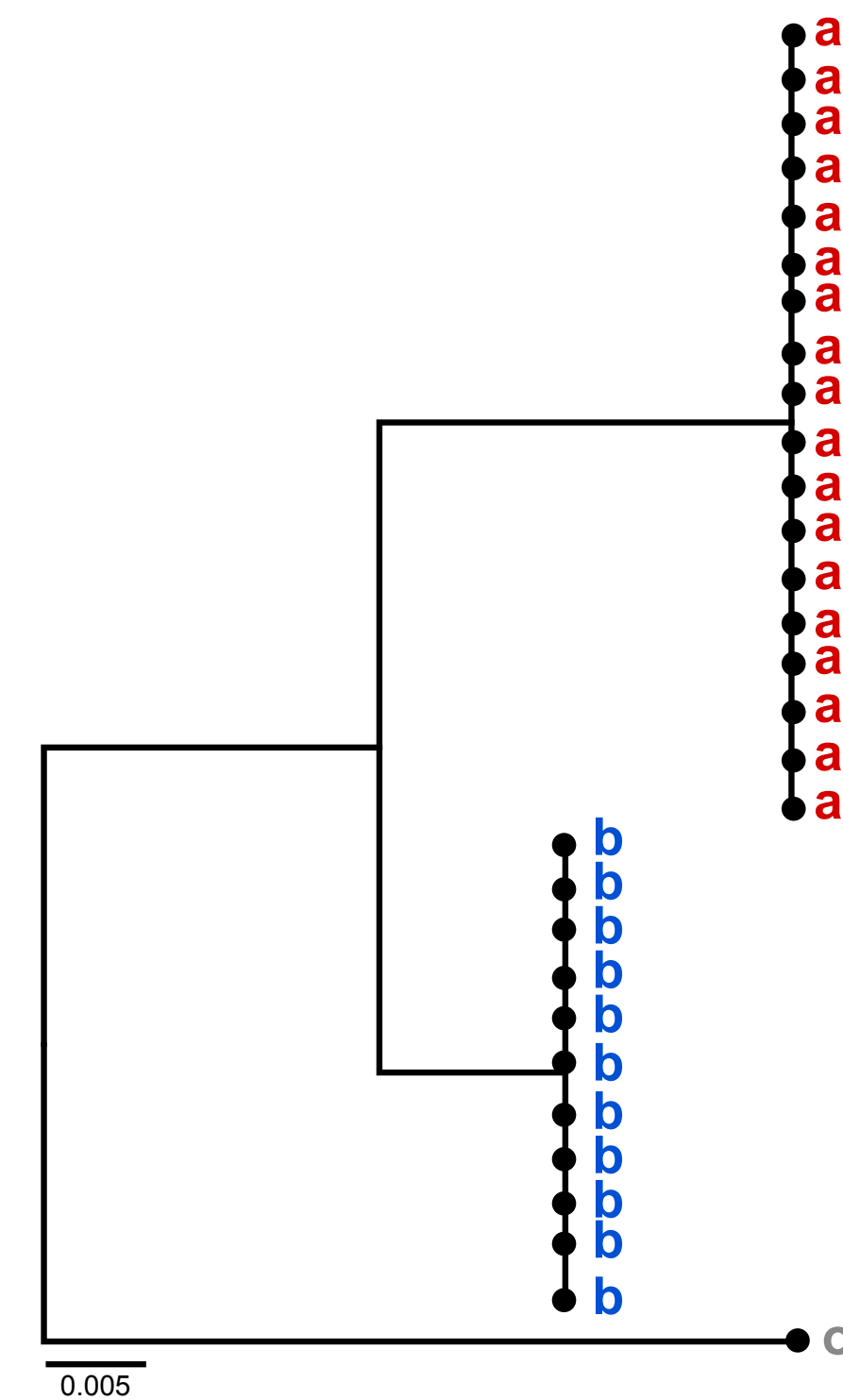**E CG14253**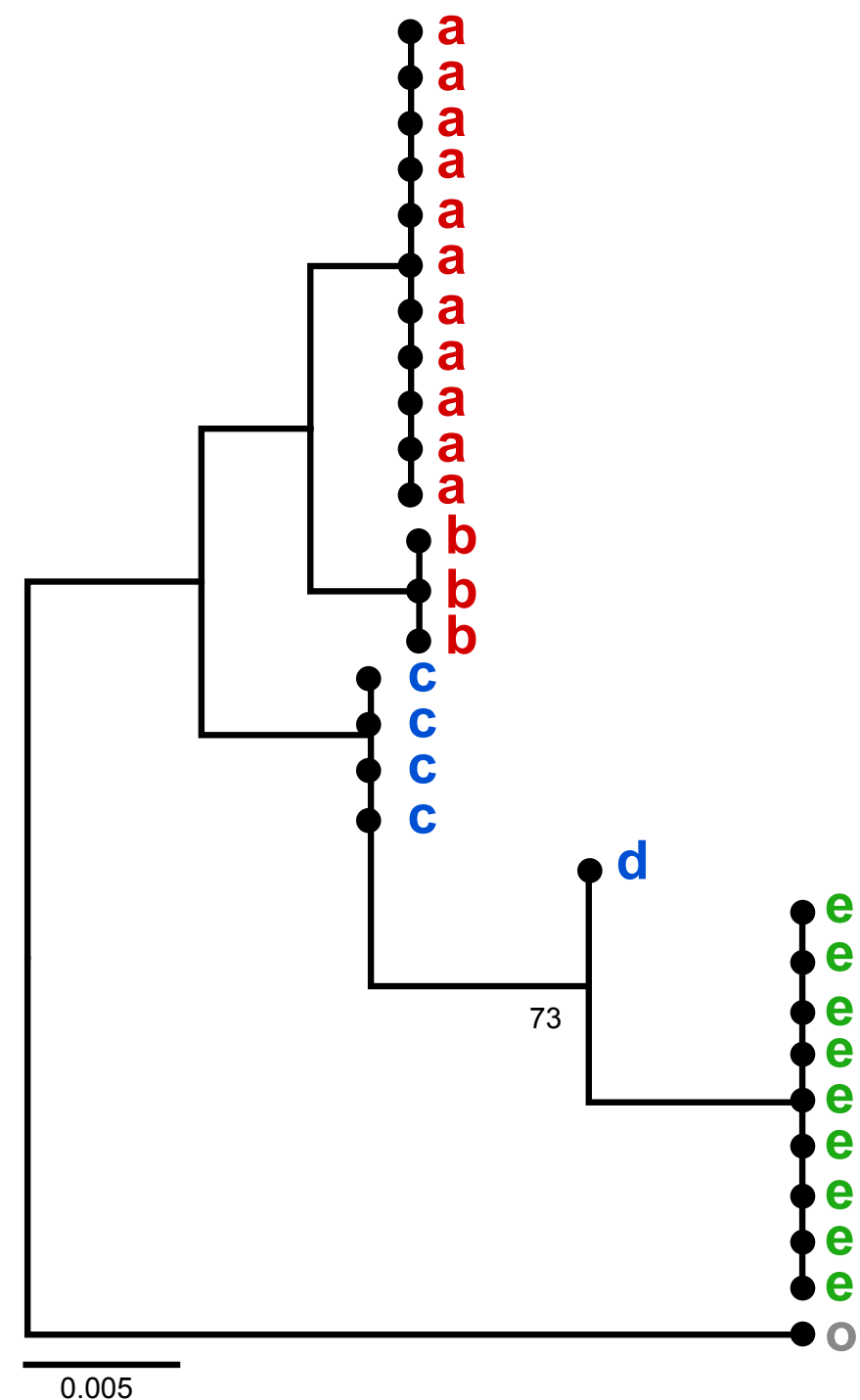**F Nmda1**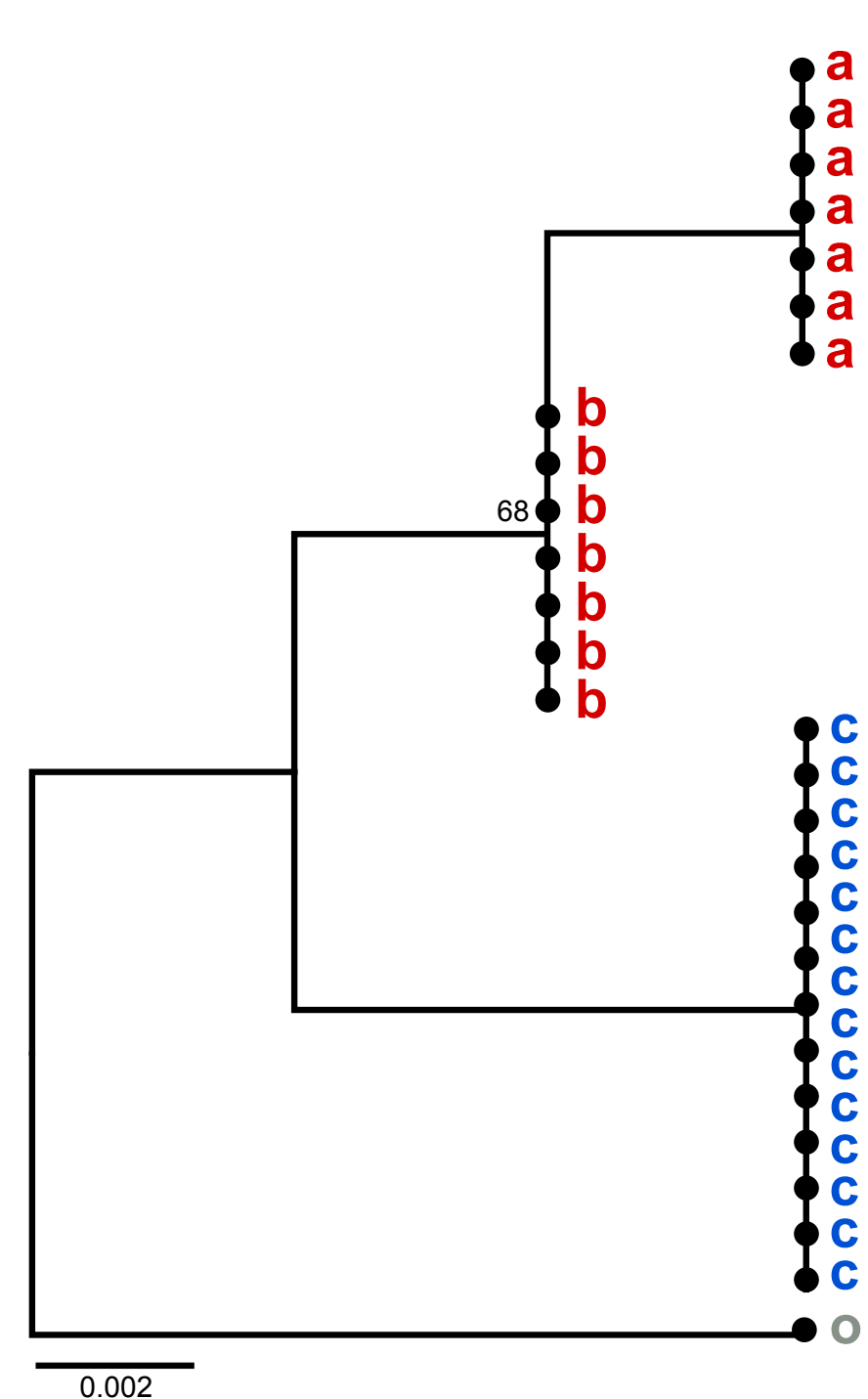**G CG6950**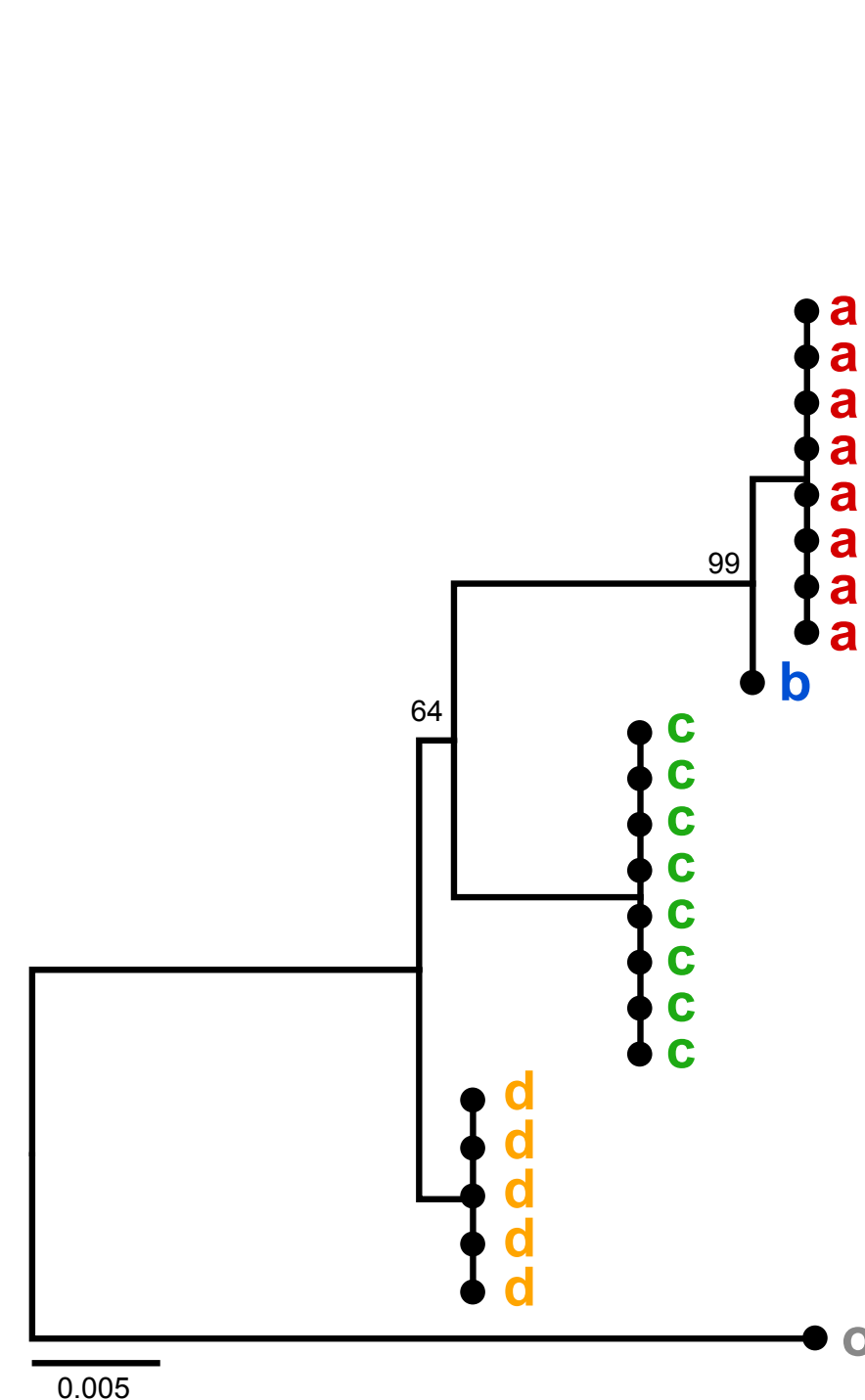**H CG10463**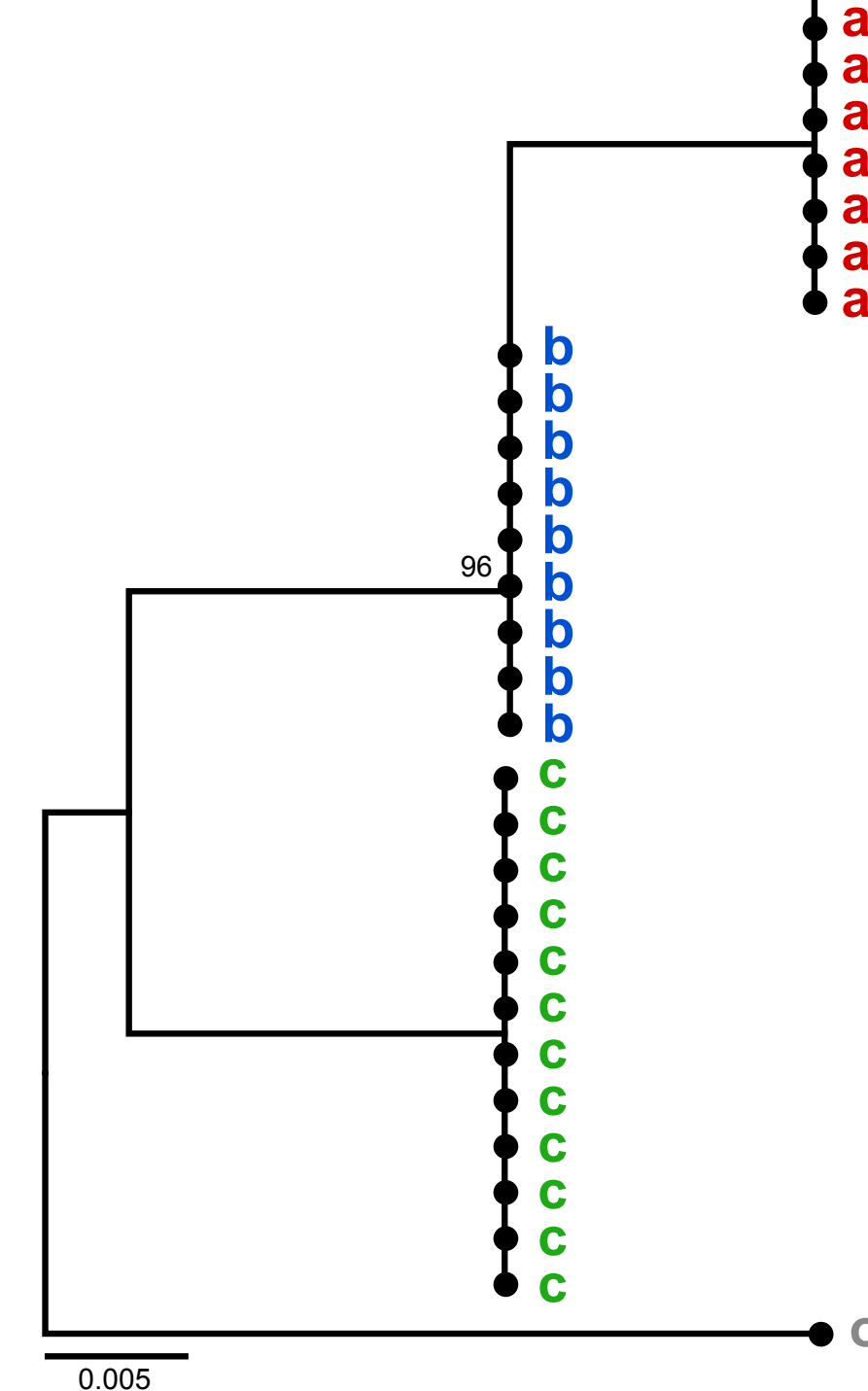

Supplement: Additional file 8: — Phylogeny of CPRs for which sequence variation could explain gene expression variation and was subject to balancing selection. Neighbor-joining trees for different alleles (a-e) of CPR are drawn for CG15743 (A), CG9044 (B), brat (C), Cyp4d1 (D), CG14253 (E), Nmda1 (F), CG6950 (G), and CG10463 (H). Drosophila simulans was used as an outgroup (o). Bootstrap values are shown for nodes with greater than 60 % support. (PDF 46 kb) [file 12862_2016_606_MOESM8_ESM.pdf]

# A CG15743

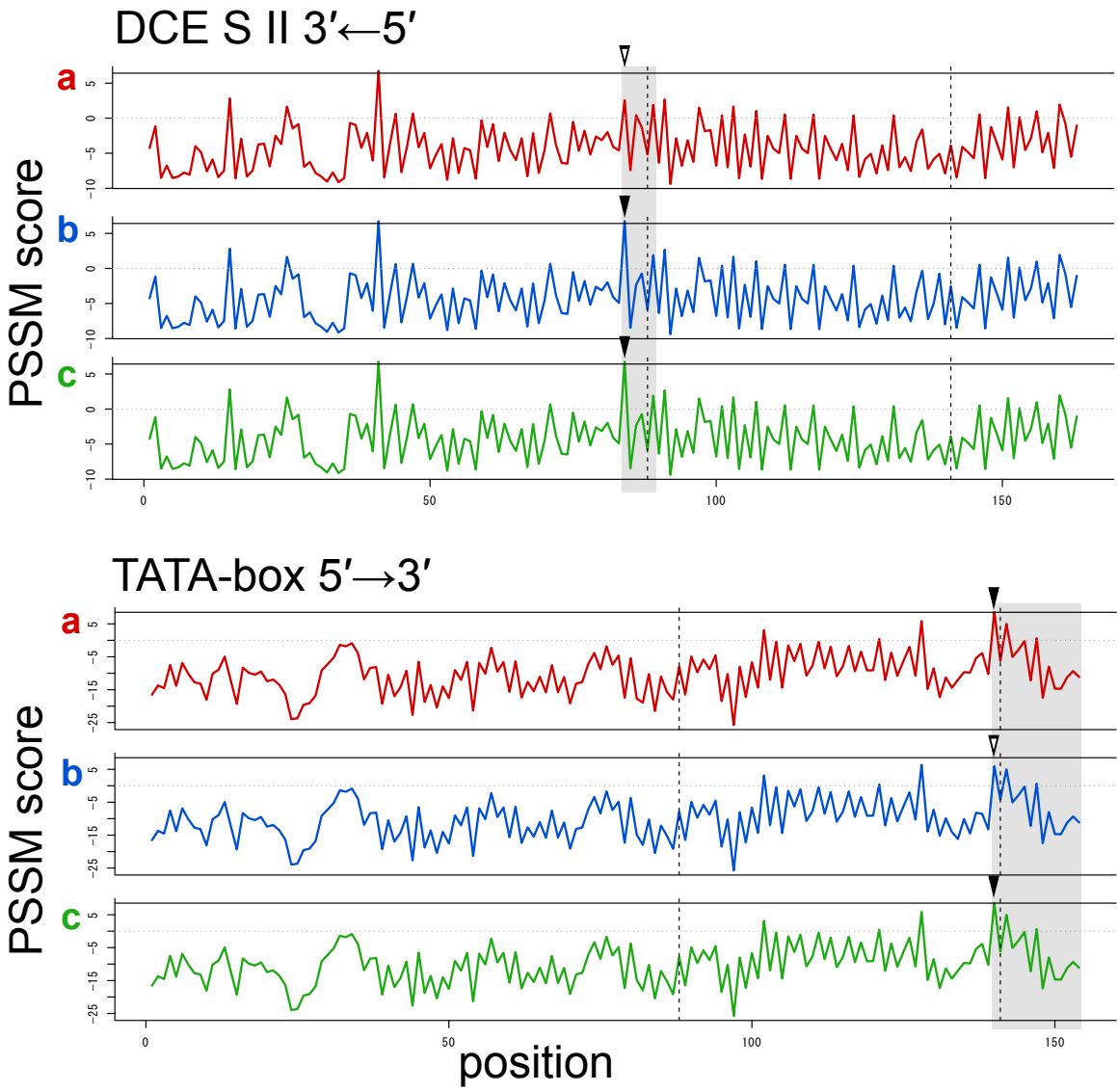

# B CG9044

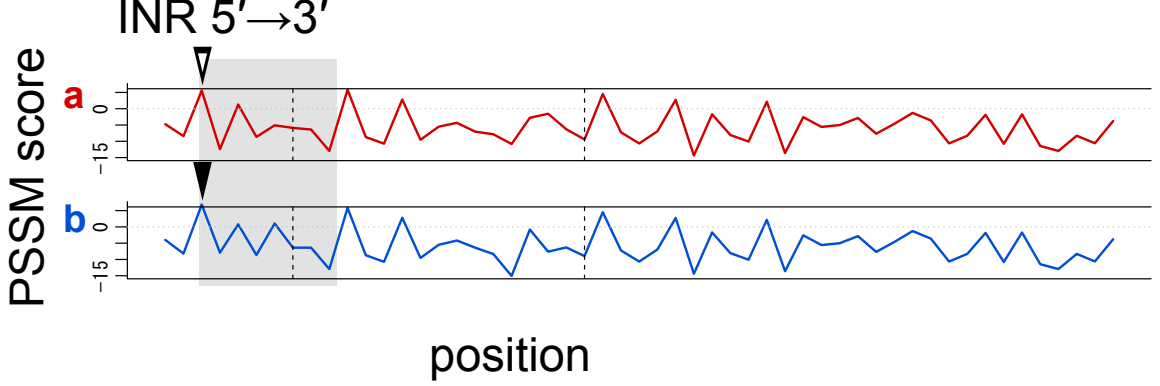

# C brat

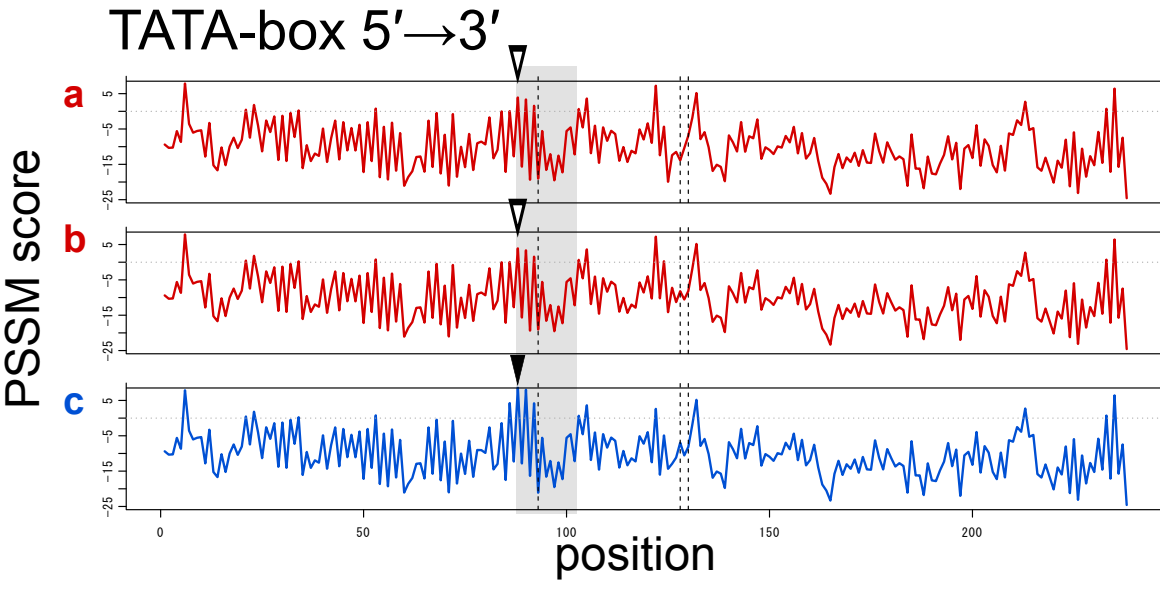

# D Cyp4d1

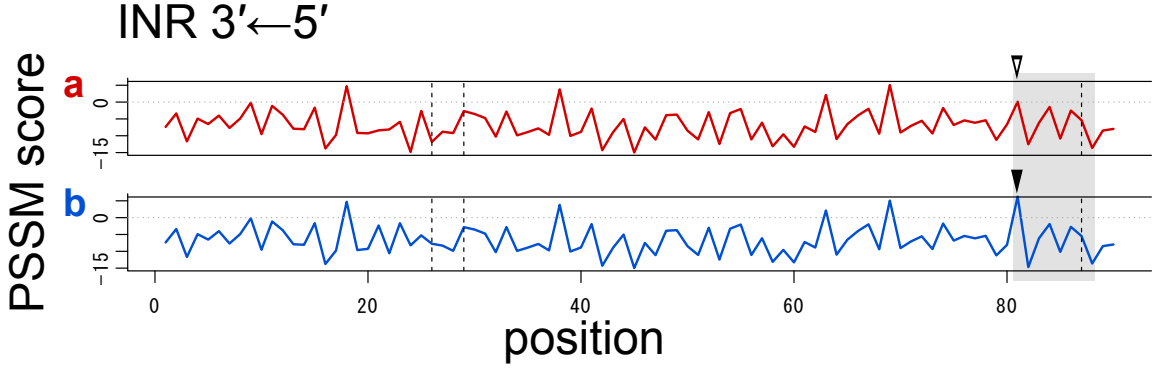

# E CG14253

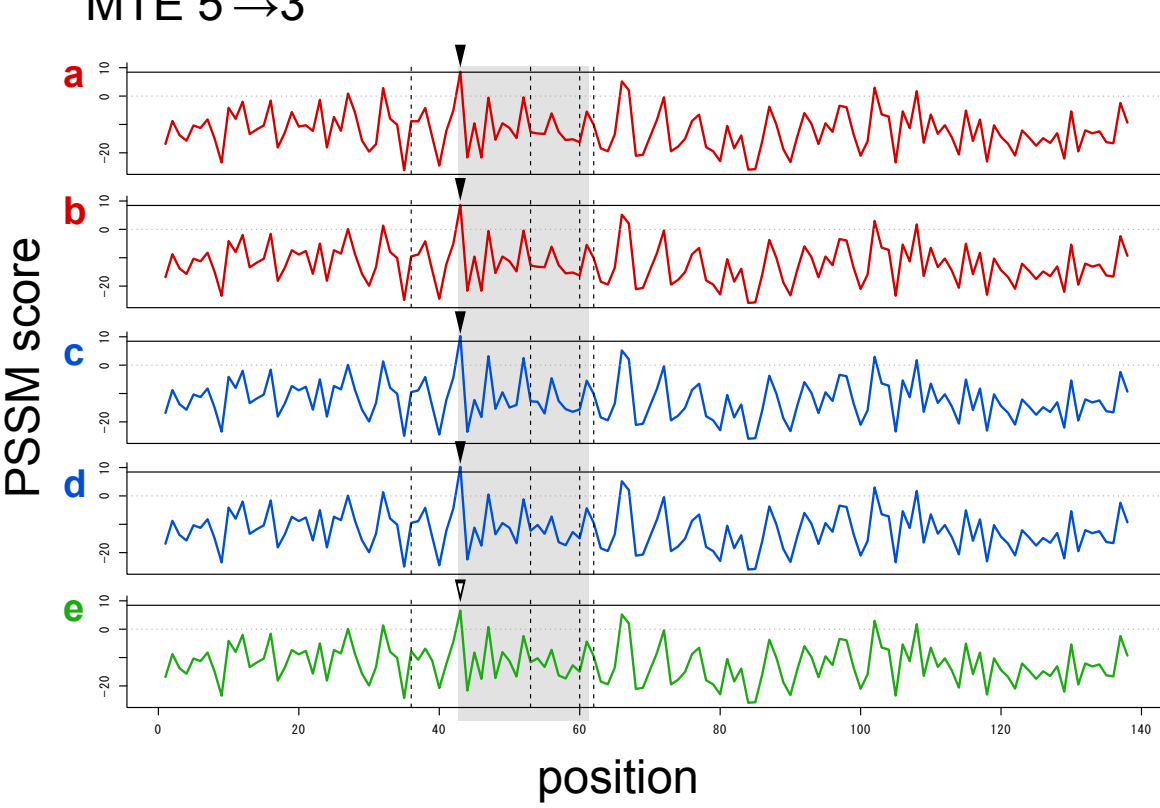

# F Nmda1

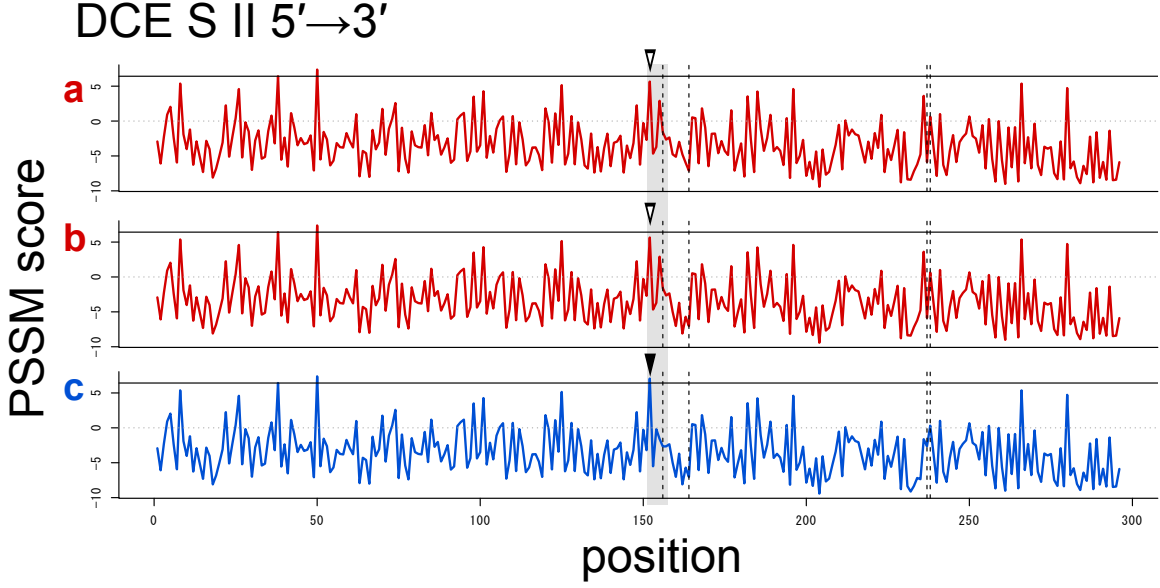

# G CG6950

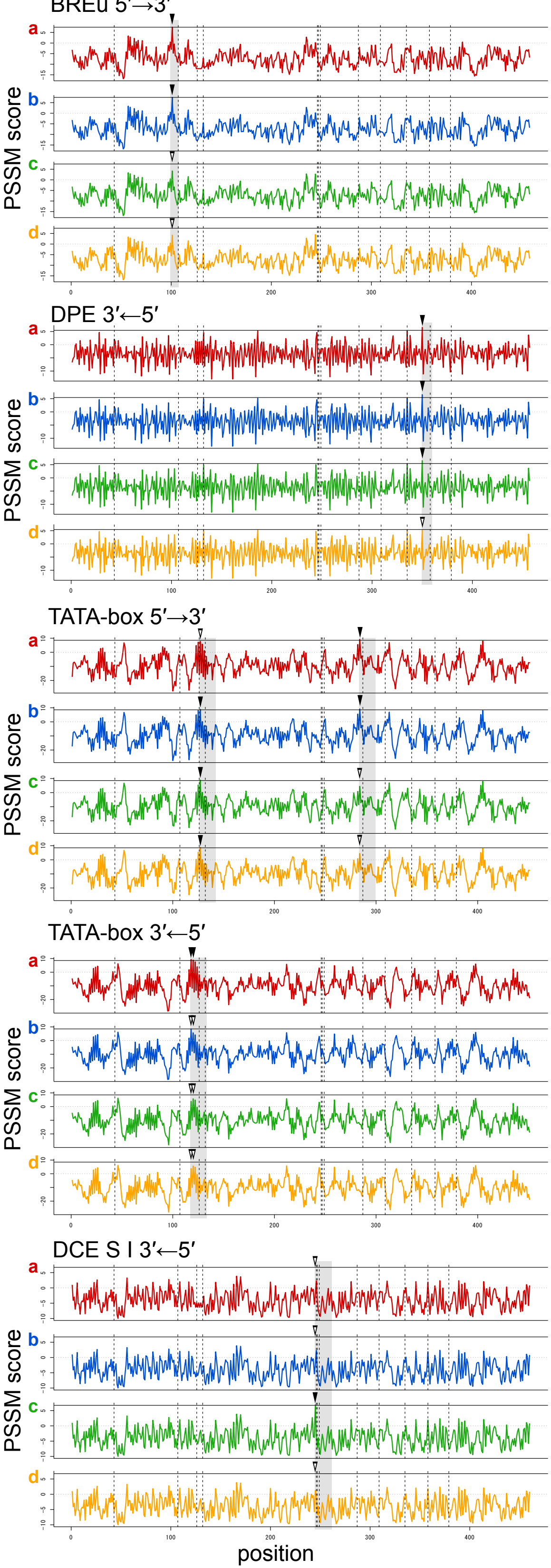

# H CG10463

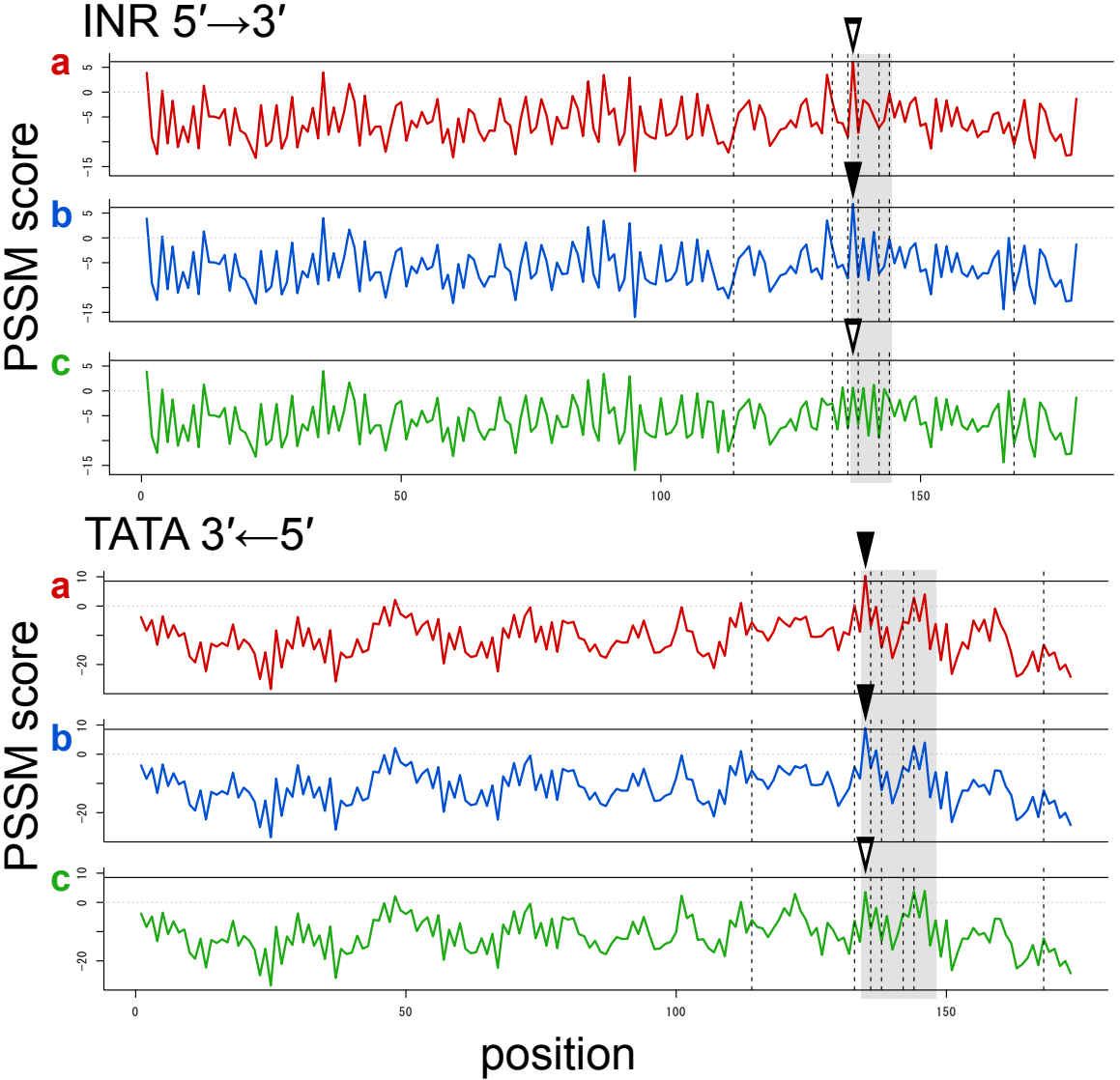

Supplement: Additional file 9: — Distribution of PSSM score along CPR sequences for which sequence variation could explain gene expression variation and was subject to balancing selection. PSSM scores (log odds finding motifs) for the binding sites of DCE S II (A and F), TATA box (A, C, G, and H), Inr (B, D, and H), MTE (E), BREu (G), DPE (G), and DCE S I (G), at all positions along CPR sequences on the strand are shown. Gray dotted horizontal lines indicate a PSSM score of zero and black horizontal lines indicate PSSM scores at threshold values above which each transcription factor is likely to bind. Black dashed vertical lines indicate the position of SNPs found in the population. Black triangles indicate positions where PSSM scores for one or more alleles were higher than the threshold value (closed triangle), while other alleles had PSSM scores lower than the threshold value (open triangle). Gray shading indicates the range of positions at which mutations affected the altered PSSM score. Different colors (red, blue, green, and yellow) indicate differences in TFBS patterns caused by sequence variation. The color and alphabet (a-e) correspond to those in Additional file 8. (PDF 200 kb) [file 12862_2016_606_MOESM9_ESM.pdf]

**A CG15743**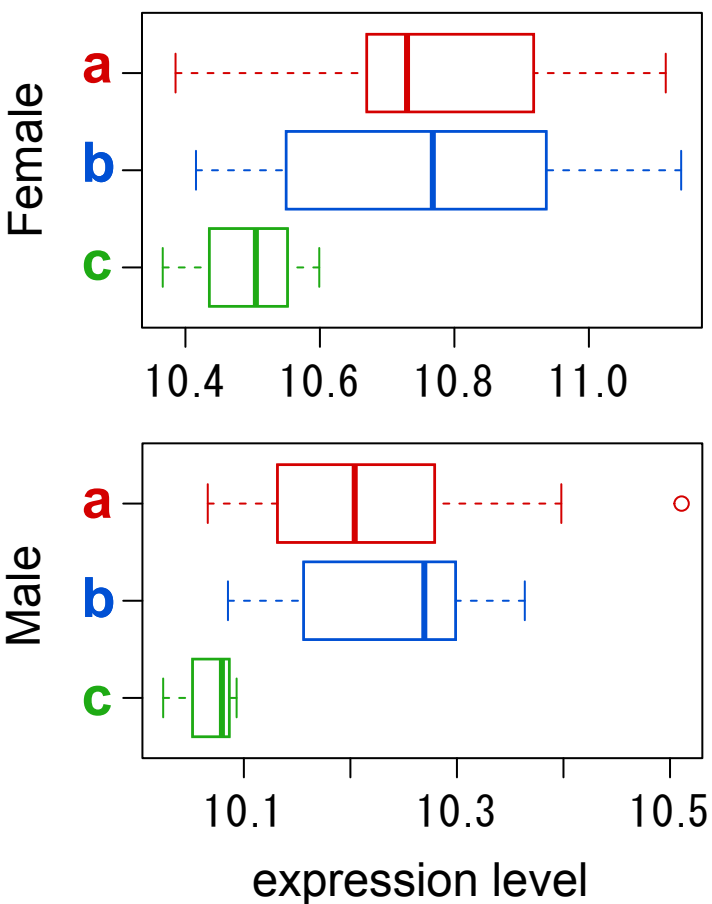**B CG9044**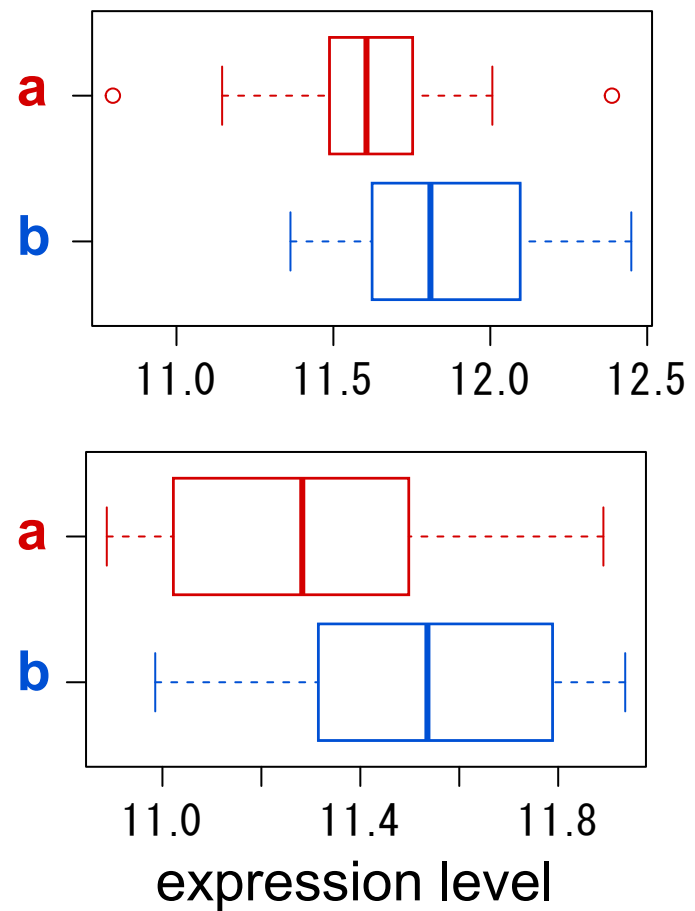**C brat**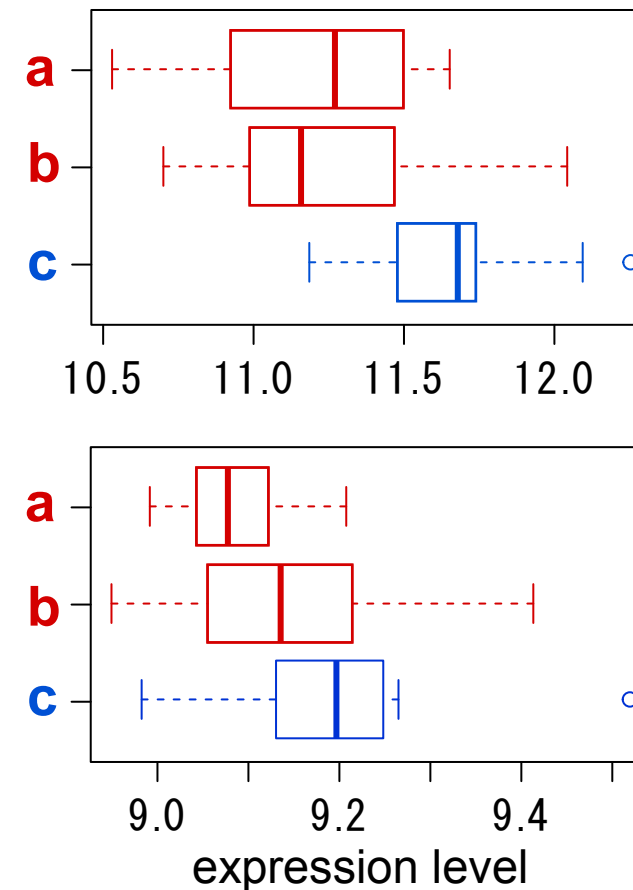**D Cyp4d1**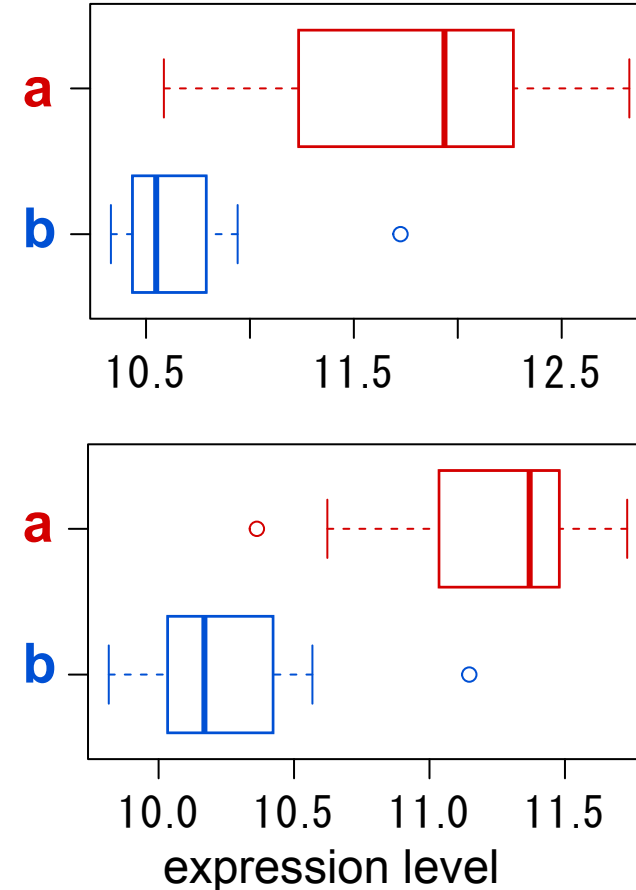**E CG14253**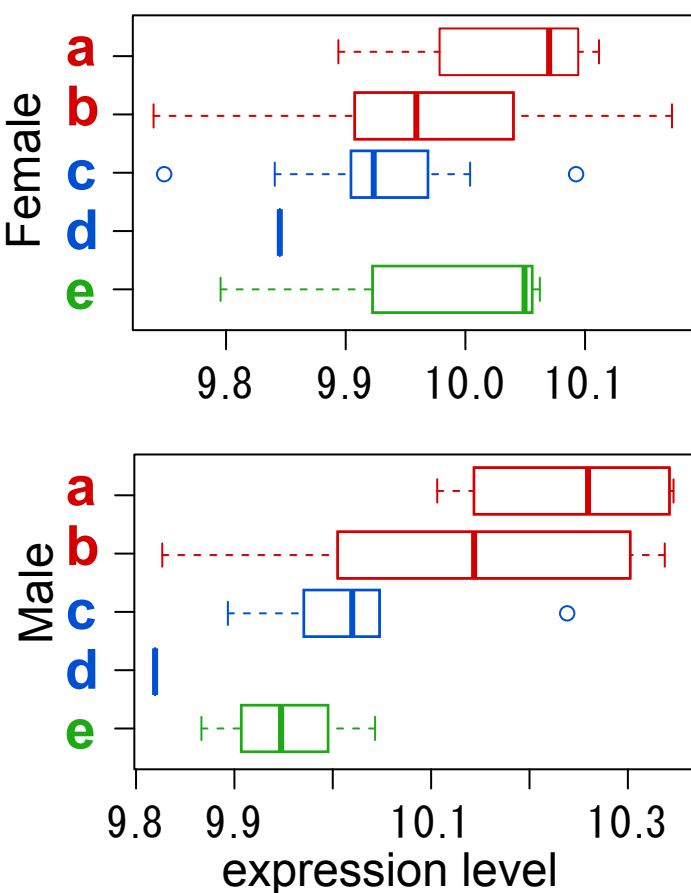**F Nmda1**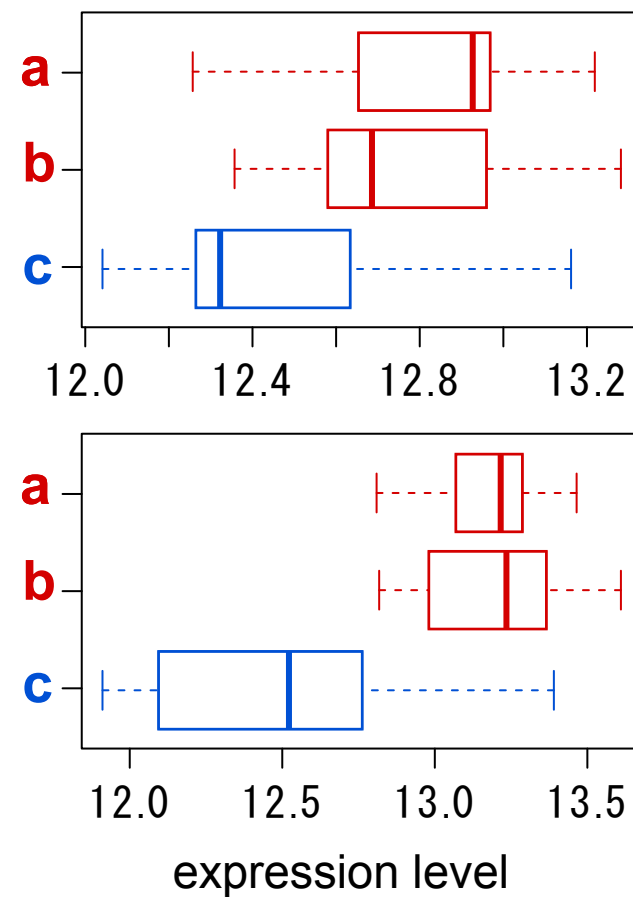**G CG6950**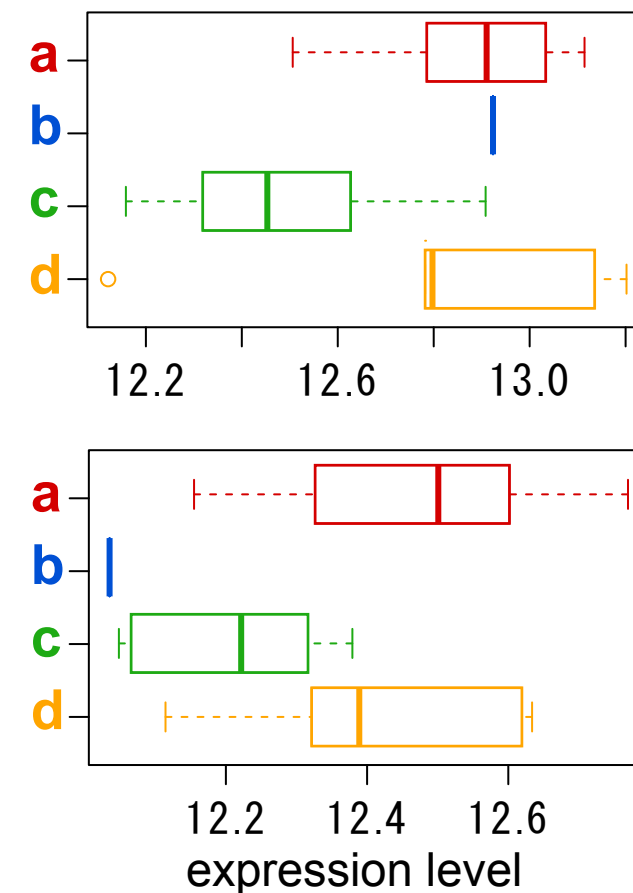**H CG10463**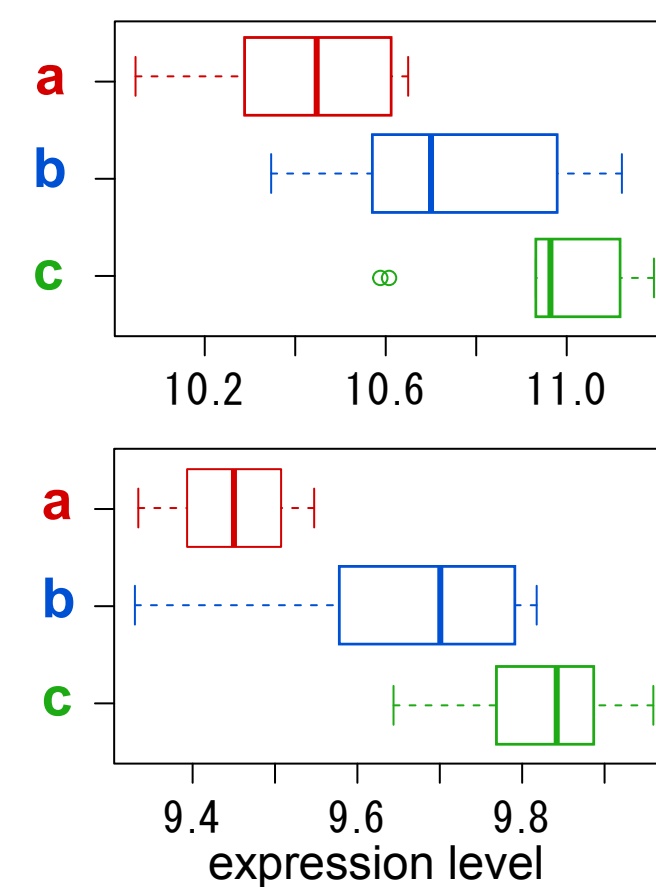

Supplement: Additional file 10: — Expression level of different alleles of CPRs for which sequence variations could explain gene expression variations and were subject to balancing selection. Expression levels of each allele were from the database using microarrays in female and male flies [16] found in a natural population for CG15743 (A), CG9044 (B), brat (C), Cyp4d1 (D), CG14253 (E), Nmda1 (F), CG6950 (G) and CG10463 (H). Color (red, blue, green, and yellow) and alphabet (a-e) correspond to those in Additional file 8. (PDF 53 kb) [file 12862_2016_606_MOESM10_ESM.pdf]
